# The Synergistic Effects of Environmental and Genetic Factors on the Regulation of Anthocyanin Accumulation in Plant Tissues

**DOI:** 10.3390/ijms241612946

**Published:** 2023-08-18

**Authors:** Van Giap Do, Youngsuk Lee, Jeong-Hee Kim, Young-Soon Kwon, Jong-Taek Park, Sangjin Yang, Juhyeon Park, Nay Myo Win, Seonae Kim

**Affiliations:** Apple Research Institute, National Institute of Horticultural and Herbal Science, Rural Development Administration, Gunwi 39000, Republic of Korea; kongfo@korea.kr (Y.L.); kimjhee@korea.kr (J.-H.K.); peat1004@korea.kr (Y.-S.K.); jongtaek@korea.kr (J.-T.P.); yangsangjin@korea.kr (S.Y.); wngus1113@korea.kr (J.P.); naymyowin@korea.kr (N.M.W.)

**Keywords:** anthocyanin biosynthesis genes, anthocyanin accumulation, apple fruit, transgenic rice calli, light stimulus, transient expression, stable expression

## Abstract

Anthocyanin accumulation is responsible for the coloration of apple fruit, and their accumulation depends on the expression of anthocyanin biosynthesis-related genes. Light is an environmental stimulus that induces fruit color by regulating genes involved in the anthocyanin biosynthesis pathway. In this study, the roles of light and genetic factors on fruit coloration and anthocyanin accumulation in apple fruit were investigated. Three genes in the anthocyanin biosynthesis pathway, *MdCHS*, *MdANS*, and *MdUFGT1*, were synthesized and cloned into a viral-based expression vector system for transient expression in ‘Ruby S’ apple fruits. Apple fruits were agroinfiltrated with expression vectors harboring *MdCHS*, *MdANS*, and *MdUFGT1*. Agroinfiltrated apple fruits were then either kept in the dark (bagged fruits) or exposed to light (exposed fruits). The agroinfiltrated fruits showed significantly different coloration patterns, transcript expression levels, and anthocyanin accumulation compared to the control fruits. Moreover, these parameters were higher in exposed fruits than in bagged fruits. For stable expression, *MdCHS* was introduced into a binary vector under the control of the rice α-amylase 3D (RAmy3D) promoter. The ectopic overexpression of *MdCHS* in transgenic rice calli showed a high accumulation of anthocyanin content. Taken together, our findings suggest that light, together with the overexpression of anthocyanin biosynthesis genes, induced the coloration and accumulation of anthocyanin content in apple fruits by upregulating the expression of the genes involved in the anthocyanin biosynthesis pathway.

## 1. Introduction

Anthocyanin is a pigment that, together with other compounds such as chlorophylls and carotenoids, is responsible for the coloration of apple fruit. The coloration patterns of apples vary depending on the amount of these compounds. Apple fruits with high coloration contribute to their quality and attract consumers’ attention, thus increasing their prospects in the fruit market. Anthocyanins have several potential health benefits owing to their antioxidant and anti-inflammatory properties [1,2], and studies suggest that they may help improve cardiovascular health by reducing the risk of heart disease and stroke [3,4,5], and may have anti-cancer properties for cancer prevention [6,7]. Moreover, the anti-inflammatory properties of anthocyanins may help reduce the risk of certain diseases, such as arthritis [8,9,10].

**Figure 1:**
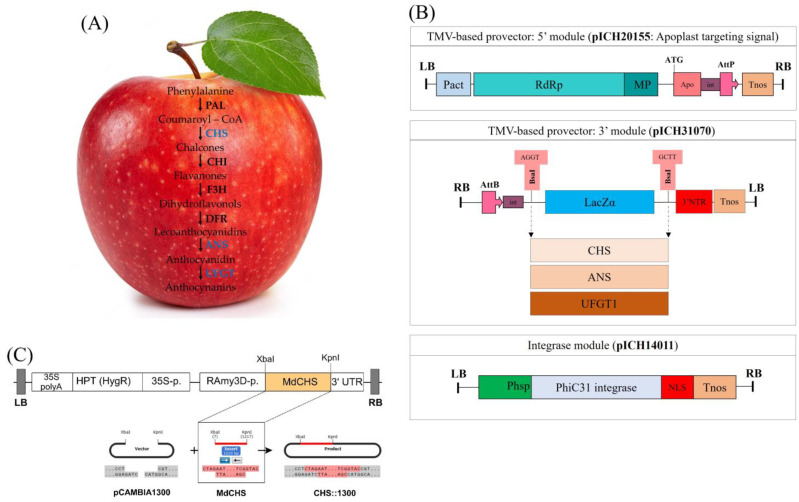
(**A**) A basic anthocyanin biosynthetic pathway. PAL, phenylalanine ammonia lyase; CHS, chalcone synthase; CHI, chalcone isomerase; F3H, flavanone 3-hydroxylase; F3′H, flavonoid 3′-hydroxylase; F3′,5′H, flavonoid 3′,5′-hydroxylase; DFR, dihydroflavonol 4-reductase; ANS, anthocyanidin synthase; UFGT, UDP-glucose: flavonoid glucosyltransferase. (**B**) Binary vectors for transient expression of *MdCHS*, *MdANS*, and *MdUFGT1*. The pICH31070 vector, a TMV-based 3′ pro-vector module carrying these three genes. pICH20155, a TMV-based 5′ pro-vector for targeting signal. pICH14011, an integrase module for recombination 5′ and 3′ pro-vectors modules. LB and RB, left and right borders; Pact, Arabidopsis actin 2 promoter; RdRp, RNA-dependent RNA polymerase; MP, movement protein; CP, coat protein; Apo, apoplast targeting presequence; int, intron; AttP and AttB, recombination sites; Tnos, nos terminator; 3 NTR, 3 nontranslated regions. (**C**) The T-DNA region of binary vectors for stable expression of *MdCHS*. RB, T-DNA right border; 3′-UTR, 3′-untranslated region of the rice α-amylase 3D gene; 35S-p, cauliflower mosaic viral (CaMV) 35S promoter; *hptII*, hygromycin phosphotransferase; 35S polyA, the terminator of 35S gene; LB, T-DNA left border.

The anthocyanin biosynthesis pathway in plants is well characterized and involves a series of enzyme-catalyzed reactions that produce anthocyanins, a group of water-soluble pigments responsible for the red, purple, and blue colors in many fruits, vegetables, and flowers [11,12]. The basic anthocyanin biosynthesis pathway is shown in Figure 1A. The pathway begins with the production of the precursor compound phenylalanine to coumaroyl-CoA by the enzyme phenylalanine ammonia lyase (PAL), followed by an enzyme-catalyzed reaction series of chalcone synthase (CHS), chalcone isomerase (CHI), flavanone 3-hydroxylase (F3H), dihydroflavonol 4-reductase (DFR), and anthocyanidin synthase (ANS), which are converted into anthocyanidins. Anthocyanidins are then catalyzed by UDP-glucose: flavonoid glucosyltransferase (UFGT) and undergo modifications, such as glycosylation or acylation, to produce the final anthocyanin pigments. The formation of anthocyanins can be regulated at various points in the biosynthetic pathway, including transcriptional regulation of genes encoding the enzymes involved in the pathway, post-transcriptional regulation of the enzymes, and regulation of enzyme activity. Genetic engineering has been used to regulate the expression of genes involved in the anthocyanin biosynthesis pathway to improve anthocyanin pigments in plants. Approaching these advances in genetic mechanisms for the regulation of pigment biosynthesis in tomato fruits has been recently reviewed [13]. Jiang et al. isolated four anthocyanin biosynthesis genes (*CHS*, *CHI*, *F3H*, and *DFR*) from eggplant (*Solanum melongena* L.) to clarify their function. Overexpression of these genes increased anthocyanin accumulation in Arabidopsis stems and siliques [14]. In another study, transient overexpression of kiwi *AcUFGT3a* increased the accumulation of anthocyanin content and its transcriptional expression level in kiwifruit (*Actinidia chinensis*) and ‘Granny Smith’ apple fruit [15]. Heterologous overexpression of litchi *LcUFGT1* increased the accumulation of anthocyanin in transgenic tobacco [16]. These results suggest that genes in the anthocyanin biosynthesis pathway play a critical role in red color formation through the accumulation of anthocyanin. In an indirect way, the accumulation of anthocyanin can also be regulated via transcription factors by binding to the promoters of genes involved in the anthocyanin biosynthesis pathway and modulating their expression. The *MYB* family of transcription factors plays a critical role in the regulation of the anthocyanin biosynthesis pathway by binding to specific DNA sequences and activating the expression of genes involved in the anthocyanin biosynthesis pathway [17,18,19,20,21]. How MBY transcription factors are involved in the regulation of anthocyanin biosynthesis has been uncovered. Zhang et al. (2019) found that the insertion of a red transposable element (redTE) upstream of the MdMYB1 promoter was associated with red coloration patterns of apple fruit. With the presence of redTE, non-red-skinned apple cultivars can still turn red under low light conditions, whereas the absence of redTE fails to produce anthocyanin effectively [22].

Light stimulus is an environmental factor that also plays an important role in regulating anthocyanin biosynthesis in plants and fruits and has been extensively reviewed [23,24,25,26]. In particular, light signals are perceived by photoreceptors such as phytochromes and cryptochromes, which then activate transcription factors involved in anthocyanin biosynthesis. These transcription factors, in turn, regulate the expression of genes that control anthocyanin production. Studies have shown that light quality and intensity can significantly affect anthocyanin synthesis in plants. Exposure to high-intensity light or far-red light can increase anthocyanin production, whereas exposure to low-intensity light or blue light reduces anthocyanin production [27,28,29,30,31]. Besides that, temperature is also an environmental factor that plays an important role in regulating anthocyanin biosynthesis in apples was studied. High temperature reduces anthocyanin concentration, and transcripts of the genes of the anthocyanin biosynthetic pathway lead to colorlessness in the skin apple of apples [32]. Contrarily, low temperature induces anthocyanin biosynthesis [32,33]. Light in combination with temperature (as multiple environmental factors) showed synergistic effects on the regulation of anthocyanin accumulation in callus cultures of red-fleshed apple and red-fleshed kiwifruit [34,35]. Thus, light triggers various physiological and biochemical processes in plants, including the regulation of anthocyanin biosynthesis.

The regulation of anthocyanin biosynthesis in plants under the influence of environmental factors such as light, temperature, hormones, nutrient availability, and genetic factors (gene overexpression) has been previously investigated. However, most studies have investigated only a single factor, either environmental or genetic. With these approaches, the previous studies focus on assessing the effects of either single or multiple environmental factors or individually studying the effect of one or more genetic factors on anthocyanin accumulation. In this study, we investigated the synergistic effects of both environmental and genetic factors on anthocyanin accumulation. We cloned three genes (*MdCHS*, *MdANS*, and *MdUFGT*) involved in the anthocyanin biosynthetic pathway to investigate their function in the regulation of anthocyanin accumulation in apple fruits and embryogenic rice calli. We also evaluated the effect of light in combination with the overexpression of these genes, focusing on coloration, transcript gene expression analysis, and anthocyanin content accumulation in apple fruits. Among the three genes that have been investigated, we found that *MdCHS* has shown to have a strong effect on the regulation of anthocyanin biosynthesis in apples and has been used to study rice calli cells further through a stable overexpression approach. These findings highlight the important role of environmental and genetic factors in controlling fruit coloration via the regulation of gene expression.

## 2. Results

### 2.1. Establishment of Expression Vectors for Transient and Stable Expression

To investigate the function of genetic factors on coloration and pigment content accumulation in apple fruit, three genes in the anthocyanin biosynthesis pathway, *MdCHS*, *MdANS*, and *MdUFGT* (Figure 1A), were synthesized and introduced into the viral-based expression vector system for transient expression. The CDS fragments of synthetic *MdCHS*, *MdANS*, and *MdUFGT1* in the packing vector pMG—Kan were digested and then introduced into the expression vector pICH31170, resulting in CHS∷31070, ANS∷31070, and UFGT1∷31070 vectors, respectively (Figure 1B). Furthermore, the *MdCHS* was also introduced into the pMYD320 vector under the control of the rice α-amylase 3D (RAmy3D) promoter for stable expression in rice callus (Figure 1C).

These expression vectors were then transformed into *A. tumefaciens* EHA105 and LBA4404 strains. The presence of the binary expression vector in transformed Agrobacterium cells was confirmed by restriction enzyme digestion (Figures S6–S9). The transformed Agrobacterial cells were inoculated for vacuum-infiltration onto the ‘RubyS’ apple fruits and for Agrobacterium-mediated transformation onto rice embryogenic callus.

### 2.2. Coloration Patterns of Apple Fruit under Light Treatment and Transient Overexpression

**Figure 2:**
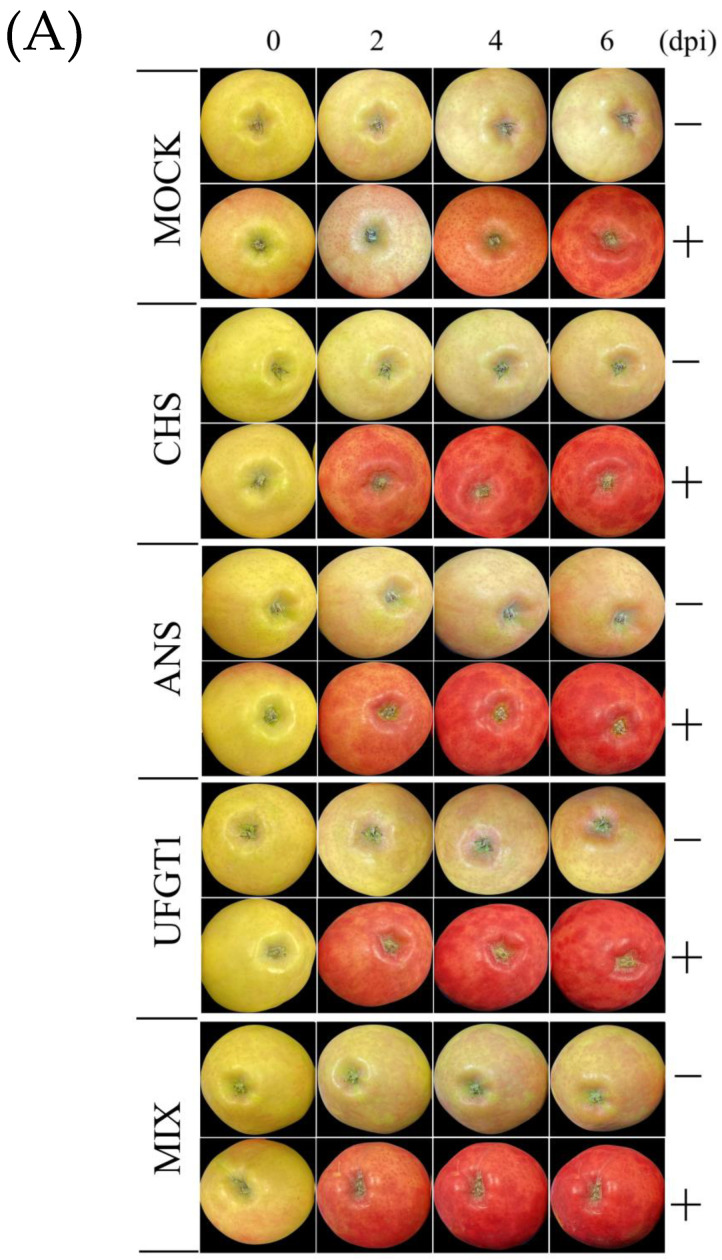

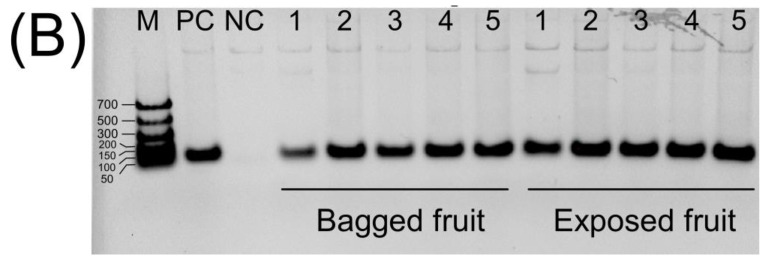
(**A**) Agroinfiltrated ‘RubyS’ apple fruits with the expression vectors using a vacuum, then treated under darkness (−) and light exposure (+), were recorded at 0–2–4–6 dpi. MOCK, fruits were agroinfiltrated with an empty vector; CHS, ANS, and UFGT, fruits were agroinfiltrated with the expression vector CHS∷31070, ANS∷301070, and UFGT∷31070, respectively; MIX, fruits were agroinfiltrated with a mixture of (CHS∷31070, ANS∷301070, and UFGT∷31070). (**B**) RT-PCR to confirm the presence of the expression vector system on apple tissues via nopaline synthase terminator (Tnos). cDNA of 6 dpi-infiltrated apple fruits was used for the detection of the Tnos. Lane M, 50 bp DNA ladder (Cat. #A702, Dyne Bio Inc. Seongnam-si, Republic of Korea); lane PC, plasmid DNA of pICH31070 vector (isolated from *E. coli*) used as positive controls; lane NC, cDNA of un-infiltrated apple fruits used as a negative control; other lanes (1, 2, 3, 4, and 5), cDNA of apple fruits which fruits were agroinfiltrated with MOCK, CHS∷31070, ANS∷301070, UFGT∷31070, and MIX, respectively.

To evaluate the effect of light and transient expression of anthocyanin biosynthesis genes on different coloration patterns of apple fruits, fruits were exposed to either light (+) or dark (−) conditions after agroinfiltration. The combination of light and transient overexpression of these genes increased fruit coloration (Figure 2A). Different light treatment conditions resulted in different coloration patterns between the two subgroups of each group. The fruits exposed to light (exposed fruits) were redder than those kept in the dark (bagged fruits). The coloration level increased in a time treatment-dependent manner, especially in the fruits that were exposed to the light. The fruits that were treated in the dark also had different coloration patterns, but not as much as those that were exposed to light. Moreover, there were also different coloration patterns among the five groups that were agroinfiltrated with different genes. The fruits of the control group (MOCK) showed less color than those of the other four groups (CHS, ANS, UFGT, and MIX). In apple, three genes from apple (*MdCHS*, *MdANS*, and *MdUFGT*) were homologous transiently expressed using a Tobacco Mosaic Virus (TMV)-based expression pro-vectors system. This expression vector system included a nopaline synthase terminator (Tnos) in all three modules, pICH31070, pICH20155, and pICH14011. Therefore, RT-PCR was conducted to elucidate the presence of the expression vector system on apple tissues using a specific primer set of Tnos (Table S2). The synthesized cDNA from RNA of infiltrated apple fruits at 6 dpi were used as templates for RT-PCR. The PCR products showed bands with expected sizes for the Tnos amplicon (84 bp), whereas no bands were detected from un-infiltrated fruits. (Figure 2B).

**Figure 3:**
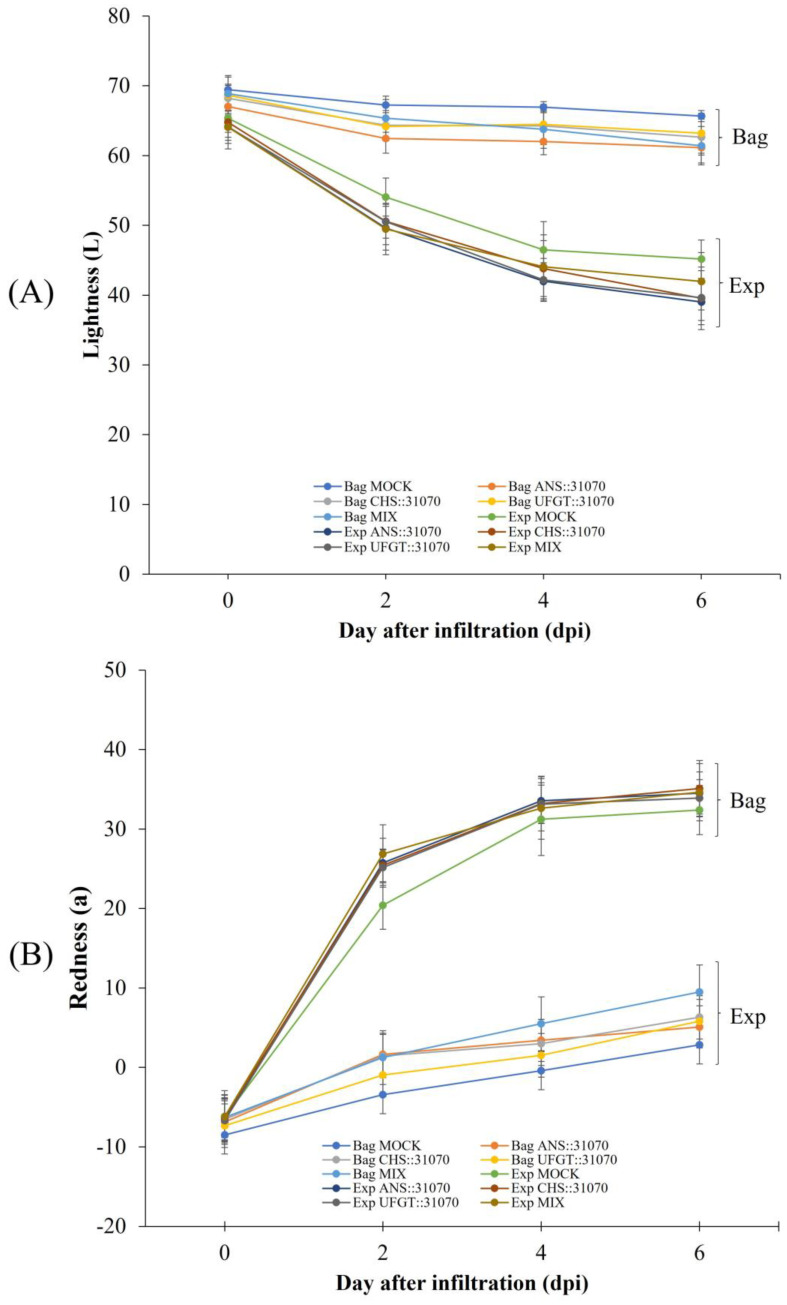

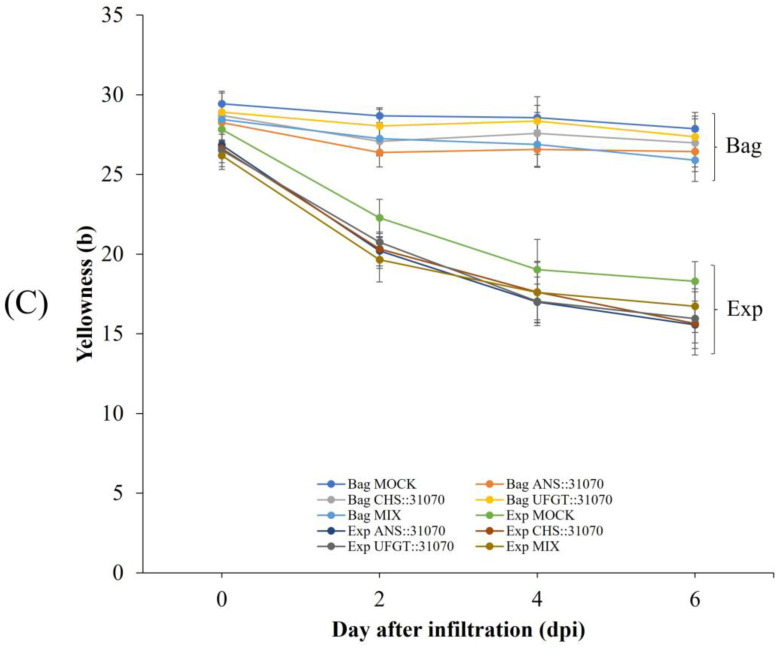
Color indices *L* (**A**), *a* (**B**), *b* (**C**) of the infiltrated fruits under different treatments of light (Bag = bagged and Exp = exposed). The color value of agroinfiltrated fruits was calculated at two-day intervals for six days. Error bars represent standard deviations (SD) (*n* = 15).

In addition, the color variables of treated fruits were measured at two-day intervals during treatment. The data showed that the color level increased during the six days of treatment from 2 to 6 days post-infiltration (dpi), expressed as Hunter values (Figure 3). Over time, the *a* (Figure 3B) value continuously increased, while *L* (Figure 3A) and *b* (Figure 3C) values decreased. Regarding fruit coloration patterns, differences in light and transient expression resulted in differences in the color values. Changes in color values were substantially different among the five groups of agroinfiltrated fruit. The *a* value of the control group was smaller than that of the other groups, resulting in less redness in both the light exposure and dark treatments (Figure 3B). Under different light treatments, all color values (*L*, *a*, and *b*) were significantly different between the light treatments in the two subgroups of the five groups. There was no significant difference in the color values at the beginning of the treatment (0 dpi) (Table S1). However, the color values quickly changed after 2 days of exposure to light (2 dpi), and then the tendency changed during the treatment. In the light treatments, even in the dark, the color values of the infiltrated fruits with expression vectors for transient expression of anthocyanin biosynthesis genes were different from those of the control fruits.

### 2.3. Expression Profiles of the Anthocyanin Biosynthesis-Related Genes in Apple Fruit

**Figure 4:**
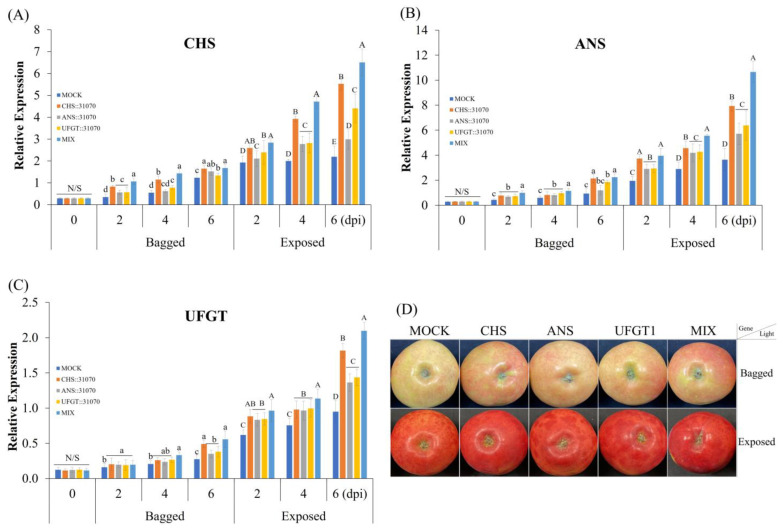
Expression profiles of anthocyanin biosynthesis genes *CHS* (**A**), *ANS* (**B**), and *UFGT1* (**C**) in the peel of ‘RubyS’ apple fruit under difference light treatment and transient expression of anthocyanin biosynthesis genes. The agroinfiltrated fruits were photographed at 6 dpi (**D**). Different lowercase and uppercase letters represent a significant difference (*p* < 0.05) in the bagged and exposed fruits, respectively. N/S refers to no significant difference (*p* < 0.05) among groups. Error bars represent the SD of three biological replicates with seven technical replicates for each biological replicate.

Next, to evaluate the effect of light and transient expression of anthocyanin biosynthesis genes on different coloration patterns of apple fruits, we analyzed the transcript expression of anthocyanin biosynthesis-related genes (*CHS*, *ANS*, and *UFGT*) by qRT-PCR. The different treatments resulted in different expression profiles of these genes (Figure 4). The expression levels of these genes were higher after light exposure than those in the fruits kept in the dark, and all genes were amplified (Figure 4A–C). Parallel to the results obtained with the coloration pattern, the expression levels of these genes were not significantly different at 0 dpi and then tended to increase from 2 to 6 dpi, especially in the exposed fruits. The expression of these genes was significantly different among the five groups under the same conditions of light treatment, with a tendency to increase in the following order: control group (MOCK) < ANS < UFGT1 < CHS < MIX group. Under both light treatment conditions, the fruits infiltrated with CHS∷31070, ANS∷31070, and UFGT1∷31070 showed that their expression was higher than that in the fruits infiltrated with the empty vector (control fruit). In correlation with differences in gene expression, the coloration patterns were also significantly different between the light treatments and slightly different among the five groups (MOCK, CHS, ANS, UFGT1, and MIX) (Figure 4D).

### 2.4. Ectopic Overexpression of MdCHS in Trangenic Rice Calli

**Figure 5:**
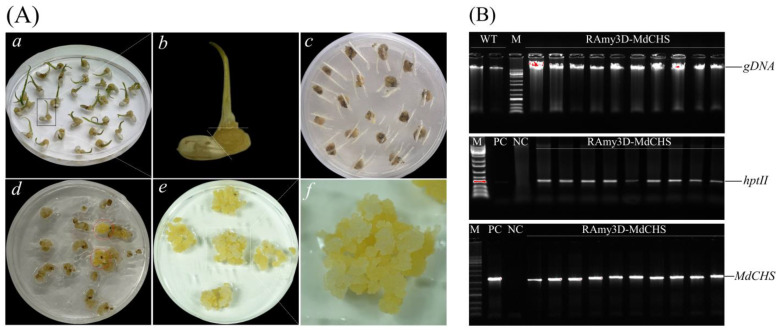
Establishment of transgenic rice calli via in vitro tissue culture using Agrobacterium-mediated transformation. (**A**) The stepwise establishment of transgenic rice. (**a**) Rice seeds (*Oryza sativa* L.) were de-husked and in vitro cultured on media for 2 weeks. (**b**) The rice embryogenic callus was detached from the germinated seedling for plant transformation. (**c**) Co-culture of rice-derived callus with transformed Agrobacteria cells. (**d**) Putative transgenic callus grown on selection media containing Hygromycin B. (**e**,**f**) Transgenic rice calli were propagated on the selection media. (**B**) Genomic DNA PCR to confirm integrating the T-DNA region of binary vectors harboring the *MdCHS* gene into rice chromosomes. Rice genomic DNA (1 µL) (upper) was used for the detection of the selection marker hygromycin phosphotransferase (*htpII*) (middle) and target gene (*MdCHS*) (below) via genomic DNA PCR analysis. Lane PC, plasmid DNA of CHS∷1300 (isolated from *E. coli*) used as positive controls; lane NC, genomic DNA of WT used as a negative control; lane M, 1 Kb plus DNA ladder (Cat. #A738, Dyne Bio Inc. Seongnam-si, Republic of Korea); other lanes (RAmy3D-MdCHS), independent transgenic lines.

Furthermore, to investigate the effect of transgenic factors on the regulation of anthocyanin accumulation in plant tissues, *MdCHS* was transformed and expressed in rice calli. Transgenic rice calli ectopic overexpressed *MdCHS* was established via in vitro tissue culture (Figure 5). Detached rice embryogenic callus were transformed with *A. tumefaciens* LBA4404 harboring the binary expression vector CHS∷1300. Numerous putative transgenic rice calli were obtained by growing on the selection media supplemented with hygromycin B (Figure 5A). The candidate of rice transgenic lines was sampled for genomic DNA extraction. Genomic DNA PCR was performed to confirm the successful insertion of the T-DNA region into the rice genome. The PCR products showed bands with expected sizes for the *MdCHS* target gene (1176 bp) and the selection marker *htpII* gene (563 bp), whereas no bands were detected from the wildtype line. (Figure 5B). Transgenic rice callus lines were propagated by subculturing every 3–4 weeks for further analyses.

### 2.5. Accumulation of Pigment Content

#### 2.5.1. Accumulation of Pigment Content in Apple Fruit

**Figure 6:**
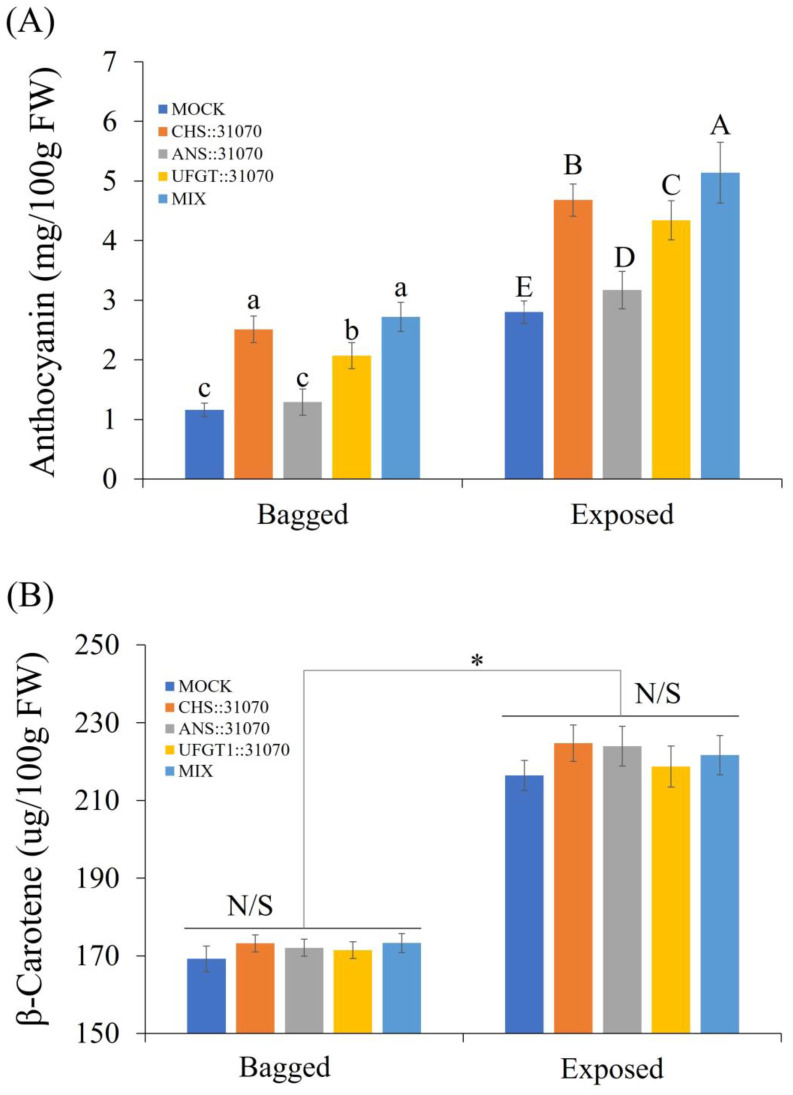
Accumulation of anthocyanin (**A**) and β-carotene contents (**B**) in the peel of ‘RubyS’ apple fruit at 6 dpi. Different lowercase and uppercase letters represent a significant difference (*p* < 0.05) in the bagged and exposed fruits for anthocyanin contents, respectively (**A**). Asterisks (*) indicate significant differences (* *p* < 0.001) from the value in the bagged and exposed fruits for β-carotene contents. N/S refers to no significant difference (*p* < 0.05) among groups (**B**). Error bars represent the SD (*n* = 15) of three biological replicates.

After evaluating the effect of the combination of light and transient expression of these genes at the transcript level, we analyzed the pigment content accumulation in treated apple fruits. Pigment content accumulation is shown in Figure 6. The accumulation of pigment in fruits exposed to light was higher than that in fruits kept in the dark. In correlation with color patterns and gene expression levels, the accumulation of anthocyanins in the exposed fruits was much higher than that in the bagged fruits. In the light exposure condition, the anthocyanin content was significantly different among the five groups in the following order: MOCK < ANS < UFGT1 < CHS < MIX. After 6 days of treatment, anthocyanin accumulation was highest in the MIX group and lowest in the MOCK group, with a value of 5.14 and 2.80 (mg/100 g FW), respectively. Under dark conditions (bagged fruits), their amounts were also significantly different, except for MOCK and ANS (Figure 5A). The amount of anthocyanin was highest in the MIX and CHS groups, followed by the UFGT1 group. The amount of β-carotene produced was also analyzed (Figure 5B). The accumulation of β-carotene in the exposed fruits was much higher than that in the bagged fruits, which was similar to anthocyanin accumulation. However, there was no significant difference between the five groups for both the exposed and bagged fruits.

#### 2.5.2. Accumulation of Pigment Content in Rice Calli

**Figure 7:**
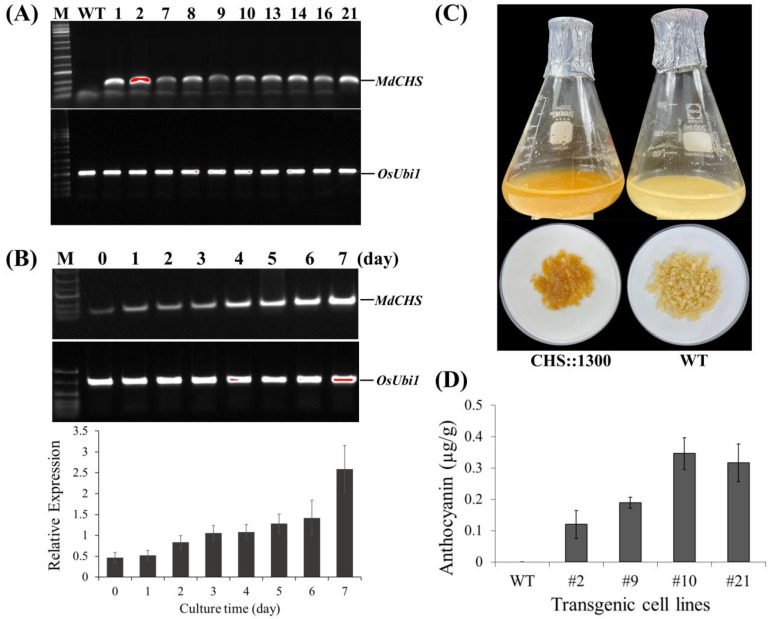
Ectopic overexpression of apple *MdCHS* gene in transgenic rice calli. (**A**) Screening high expression cell lines via RT-PCR. (**B**) Time course expression over a period of 7 days induction under sucrose starvation on a solid medium (without sucrose) via RT-PCR (upper) and qRT-PCR (lower). (**C**) Transgenic rice cells cultured on suspension medium and (**D**) their anthocyanin accumulation at 7 dpi under sucrose starvation. The expression levels of *MdCHS* in (**A**,**B**) were normalized to a rice reference gene *OsUbi1*. Lane M, 1 Kb plus DNA ladder marker; lane WT, wildtype lines; other lanes, independent transgenic lines of CHS∷1300. Error bars represent the SD of three biological replicates.

After establishing transgenic rice calli for stable expression, we first screened the transgenic cell lines with high transcript expression levels. Ten cell lines were selected for evaluation of transcript expression level via RT-PCR. Gene expression levels are different among cell lines (Figure 7A). To investigate the dynamic transcript expression level of *MdCHS*, time course induction was conducted. Cell line #2 was induced on the induction media (solid media without sucrose) for a 7-day culture period, and rice cells were sampled at one-day intervals for quantification of gene expression. As the results showed in Figure 7B, the expression level of *MdCHS* was detected early and steadily increased in correlation with induction time. For anthocyanin accumulation analysis, the transgenic rice cell suspension was cultured and then induced on the liquid induction media for 7 days (Figure 7C). After the 7-day induction period, the rice cells were collected and vacuum-dried for anthocyanin content analysis. In all transgenic lines, the accumulation of anthocyanins was induced. The anthocyanin contents accumulated highest in cell line #10 with 0.346 µg/gFW and lowest in cell line #2 with 0.120 µg/gFW. In contrast, there are no anthocyanins accumulated in the wildtype lines that can be detectable (Figure 7D). These results suggest that anthocyanin contents in the transgenic rice cells were induced by heterologous overexpression of *MdCHS*.

### 2.6. Pearson’s Correlation Coefficient Analysis

**Figure 8:**
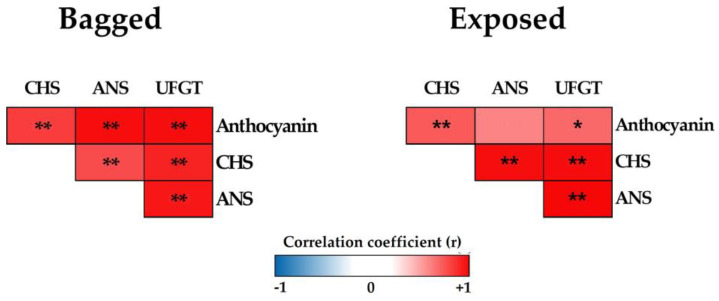
Correlation analysis of anthocyanin-related gene expression levels and the accumulation of anthocyanin in apple fruits at 6 dpi. The correlation coefficient varied as indicated by the level colored. Red refers to positive correlations, and blue refers to negative correlations with the level of significance * *p* < 0.05, ** *p* < 0.01.

Pearson’s correlation coefficient analysis was performed to evaluate the association between anthocyanin contents and gene expressions of each treatment for apple fruits (Figure 8). In the bagged treatment, anthocyanin accumulation was positively correlated with the expression of all three genes. For exposed treatment, there was also a positively correlated between anthocyanin contents and gene expressions. However, the bagged fruit showed a strongly correlated than exposed fruit. Correlation coefficients were also significantly greater in the bagged fruit than in exposed fruit, especially in ANS and UFGT. In general, the correlation coefficients analysis showed that there was a positive correlation from a strong to very strong correlation level between anthocyanin accumulation and the expression levels of anthocyanin-related genes, except for ANS, which was intermediate.

## 3. Discussion

In this study, different treatments resulted in different coloration patterns, gene expression levels, and pigment content accumulation in apple fruit. First, we observed differences in the coloration patterns across the five groups under different transient expressions of anthocyanin biosynthesis genes (MOCK, CHS, ANS, UFGT1, and MIX) and the two subgroups under different light treatments (dark and light exposure) (Figure 2 and Figure 3). Under light-exposure conditions, the exposed fruits were redder than the fruits treated in the dark. Similar phenomena were observed in a previous study [36]. It has been reported that fruits under dark conditions lack red color compared to those exposed to light. Other studies using light to control fruit coloration have been carried out on apples. Blocking of light by bagging apple fruit inhibited the coloration of bagged fruit, leading to colorlessness, but re-exposure of the bagged fruits to light quickly turned the fruit red in color [37]. Enhancing light in apple fruit by reflected light stimulates the color in apples [31]. Besides light quantity, light quality (spectral wavelength) also affects fruit coloration and anthocyanin accumulation. Ryu et al. found that UV-B treatment within 3 days stimulated anthocyanin accumulation and upregulated the gene expression of the flavonoid-related genes, including transcription factor, compared to other light exposure in ‘Fuji’ apple skin [38]. To examine the effect of transgenic factors on apple fruit coloration, the transient expression of anthocyanin biosynthesis genes was examined using a viral-based expression vector system (Figure 1B). This vector expression system has the advantage of rapid replication of insert genes because a part of them is constructed by recombining from viral mRNA replicon [39,40]. Using this vector expression system allows rapid and high-yield production of recombinant protein in plants [41,42]. The fruits infiltrated with anthocyanin biosynthesis genes were much more colorful than the control (MOCK) fruits (Figure 2 and Figure 3). The anthocyanin biosynthesis pathway includes a series of structural enzymes encoded by their responsible genes. Therefore, introducing these genes into apple tissue cells can upregulate the anthocyanin biosynthesis pathway, resulting in the production and synthesis of additional downstream bioproducts, thus upregulating anthocyanin contents and stimulating apple fruit coloration.

Moreover, we analyzed the expression of three anthocyanin biosynthesis genes at the transcript level. All transcripts increased gradually from 2 to 6 dpi after agroinfiltration. Fruits exposed to light showed a higher expression level than fruits kept in the dark (Figure 4). Similarly, it has been reported that the expression of *CHS*, *ANS*, and *UFGT* in bagged fruits was lower than that in non-bagged fruits [43]. We previously observed that the expression of anthocyanin synthesis-related structural genes in the exposed fruit was much higher than in the bagged fruit in two apple cultivars, ‘Arisoo’ and ‘Summer Prince’ [31]. Kim et al. found that the gene expression and anthocyanin accumulation were induced by light exposure after bag removal in the skin of the ‘Fuji’ apple fruit [12]. Moreover, light also stimulates coloration and induces anthocyanin biosynthesis in apple fruit by modulating the expression of transcription factors such as MYB1, MYBA, and MYB10 by binding to their promoter [44,45,46,47]. Additionally, we also analyzed the transcript expression of three anthocyanin biosynthesis genes in *N. benthiana*. Tobacco leaves were infiltrated with CHS∷31070, ANS∷31070, and UFGT1∷31070 vectors. Infiltrated showed the high expression of these genes was observed in tobacco leaves (Figure S10).

Using three marker genes, *CHS* (Figure 4A), *ANS* (Figure 4B), and *UFGT* (Figure 4C), we evaluated their expression levels across five groups of agroinfiltrated apple fruit. As shown in Figure 4, in accordance with the coloration patterns, the gene expression levels within the five treatment groups exhibited tended to increase from 2 to 4 dpi and reached the highest at 6 dpi for both light treatments. There was no significant difference among groups observed at 0 dpi, as expected, since the fruits were well-selected in uniform quality among groups before initiating treatments (Table S1). Notably, apples infiltrated with CHS∷3170 showed a significant upregulation than other gene treatments (ANS∷3170 and UFGT∷3170) in both light treatments, especially at 6 dpi. There was not much or less significant difference in the gene expression of apples infiltrated with ANS∷3170 and UFGT∷31700. This suggests that the expression of the CHS gene could be more induced than other genes in the anthocyanin biosynthesis pathway in our study. Additionally, the correlation coefficients between anthocyanin accumulation and gene expression levels also showed that there was a strong positive correlation in the infiltrated fruits with CHS∷31070 than that of the others in the exposed fruit (Figure 8). Kondo et al. (2002) showed that the expression of *CHS*, *ANS*, and *UFGluT* was higher than other genes (*F3H* and *DFR*), and their expression was also upregulated in the non-bagged fruits but not in bagged fruits of ‘Tsugaru’ at 98 DAFB. However, in the same study, the authors also showed that together with *CHS* and *UFGT*, the expression of *F3H* was upregulated, while *ANS* exhibited low expression at 20 DAFB [43]. It shows that the expression level of these genes is not only light-dependent but also depends on fruit development stages.

Finally, the accumulation of anthocyanin in the exposed fruits was much higher than that in the bagged fruits, and its accumulation in fruits infiltrated with anthocyanin biosynthesis genes was significantly different from that in control fruits, in correlation with color patterns and gene expression levels. This indicates that the combination of light and transient overexpression of anthocyanin biosynthesis upregulates their expression levels, induces pigment content, and stimulates the color of the ‘RubyS’ apple fruit. In other studies, apple fruit re-exposure to sunlight after bag removal showed much more colorization and anthocyanin amounts [48,49].

The results obtained from the apple fruits with these differences were more dependent on environmental factors than genetic factors, suggesting that light plays a vital role in the determination of skin coloration in apple fruit in addition to the preliminary results obtained in this study on the improvement of fruit color using light and the transient overexpression of specific genes. However, the influence of genetic factors is less effective than that of light, suggesting future studies using stable overexpression instead of transient overexpression. Therefore, to evaluate the role of transgenic factors on the regulation of anthocyanin accumulation in plant tissues, *MdCHS* was heterologously expressed in rice calli for the stable expression system study (Figure 6). The apple *MdCHS* gene was introduced into the binary vector driven by the rice α-amylase 3D (RAmy3D) promoter. RAmy3D is an inducible promoter that is strongly expressed under sugar starvation. This vector system has been widely used for the production of recombinant proteins in rice cell suspension culture [50,51,52,53]. In this study, the ectopic overexpression of the apple *MdCHS* gene in rice calli led to the induction of anthocyanin accumulation (Figure 7). The findings highlight the potential of using this transgenic approach to manipulate pigment biosynthesis in rice and potentially other crops.

## 4. Materials and Methods

### 4.1. Plant Materials

#### 4.1.1. Apple Fruit

The fruit of the ‘RubyS’ apple at harvesting stages was collected, and only fruits with the same quality (size, weight, coloration, and without disease symptoms) were carefully selected for further treatments. The selected fruits were divided into several groups according to different treatments, and the characteristics of the fruits with uniform size, weight, and quality among groups are shown in Table S1.

#### 4.1.2. Rice Embryogenic Calli In Vitro Culture and Rice Cell Suspension Culture

Rice seeds (*Oryza sativa* L. Samkwang) were used as the source material for callus induction. Dehulled rice kernels with normal size and intact embryos were carefully selected. The kernels were initially washed in 70% ethanol with shaking for 2–5 min and then rinsed with sterile water. For surface sterilization, the kernels were soaked with a half-strength of NaOCl, supplemented with 0.01% Tween20 for 15 min with shaking. After several rinses with sterile water, excess water was blotted from the kernels using autoclaved Whatman filter paper before seeding them onto callus induction medium plates. Subsequently, 15–20 sterilized kernels were placed on callus induction medium plates N6CI (4 g/L of N6 medium salts (Duchefa, Haarlem, The Netherlands), 30 g/L of sucrose, and 2 mg/L of 2,4-dichlorophenoxyacetic acid (2,4-D), 0.02 mg/L of kinetin, and 2.3 g/L of phytagel, pH 5.7) and culture as previously described [54].

To obtain rice cell suspension culture for heterologous expression of anthocyanin, the transgenic rice cell was cultured in a 300-mL Erlenmeyer flask filled with 50 mL N6 medium as previously described [51].

### 4.2. Construction of Plant Expression Vectors

Sequence information for *MdCHS* (AB074485.1), *MdANS* (AF117269.1), and *MdUFGT1* (AF117267.1) was obtained from the NCBI database (https://www.ncbi.nlm.nih.gov/, accessed on 23 July 2021). The coding sequence (CDS) of the three anthocyanin biosynthesis-related genes, *MdCHS* (1176 bp), *MdANS* (1074 bp), and *MdUFGT1* (1452 bp), are shown in Figure S1. The CDS of these genes was synthesized by Macrogen Co., Ltd. (Seoul, Republic of Korea). For cloning *MdCHS*, *MdANS*, and *MdUFGT1* into expression vectors, BsaI-XbaI-BamHI and BsaI-Acc65I/KpnI restriction enzyme sites were added to the 5′– end and 3′– end of these gene sequences, respectively (Figure S1). Then, these genes were subcloned into the packing vector pMG—Kan (Figure S2), yielding vectors CHS—pMG—Kan (Figure S3), ANS—pMG—Kan (Figure S4), and UFGT1—pMG—Kan (Figure S5).

For transient expression, a Tobacco Mosaic Virus (TMV)-based expression pro-vectors was used. This expression vector system comprises three modules (unit vectors): pICH31070, pICH20155, and pICH14011. pICH31070 is a TMV-based 3′ pro-vector module for cloning the gene-of-interest (GOI). pICH20155 a 5′ pro-vector module necessary for targeting the expressed GOI to apoplasts due to the translational fusion with an apoplast-targeting signal peptide (rice alpha-amylase endoplasmic reticulum-targeting peptide). pICH14011 is an integrase module encoding PhiC31 integrase and in charge of recombination 5′ and 3′ pro-vectors modules resulting in a completely functional expression vector system for transient expression in planta [55]. For transient expression analysis of *MdCHS*, *MdANS*, and *MdUFGT* using this expression vector system, these genes were cloned into the plant expression vector pICH31070. The packing vectors CHS—pMG—Kan, ANS—pMG—Kan, and UFGT1—pMG—Kan was cut with the BsaI (Cat. #ER0292; Thermo Fisher Scientific, Waltham, MA, USA) at room temperature for 2 h. The *MdCHS*, *MdANS*, and *MdUFGT1* fragments were eluted and then ligated into the same restriction enzyme sites in the vector pICH31070 using the T4 DNA ligase enzyme (Cat. #2011B; Takara, Kusatsu, Japan). For stable expression (ectopic overexpression in rice callus), the *MdCHS* gene was cloned into a binary vector pMYD320 [51] using XbaI-KpnI restriction enzyme sites.

The ligation mixture was transformed into *Escherichia coli* DH5α competent cells (Cat. #9027, Takara, Kusatsu, Japan) according to the manufacturer’s instructions. After transformation, *E. coli* DH5α was cultured on the LB agar medium plus 50 μg/mL kanamycin (Cat. #DF0445-17-4, BD Difco™, Franklin Lakes, NJ, USA) at 37 °C overnight. The transformed *E. coli* DH5α colonies were inoculated into 5 mL LB broth medium (Cat. #DF0446-17-3, BD Difco™) containing antibiotics and cultured in a shaking incubator at 37 °C and 180 rpm overnight. The success of constructed expression vectors was confirmed by BsaI and XbaI-KpnI enzyme digestion.

### 4.3. Agrobacterium Transformation

The plasmid DNA of expression vectors pICH31070 harboring *MdCHS*, *MdANS*, and *MdUFGT1* genes isolated from *E. coli* DH5α using a plasmid DNA purification kit (iNtRON MEGAquick-spin™ Plus, Seongnam, Republic of Korea). Binary expression vectors were transformed into *Agrobacterium tumefaciens* EHA105 competent cells using the freeze–thaw method [56]. The plasmid DNA of binary vector CHS∷1300 was transformed into *A. tumefaciens* LBA4404 by the triparental mating method [57]. The presence of the binary expression vector in transformed Agrobacterium cells was confirmed by enzyme digestion.

### 4.4. Plant Tissue Transformation

#### 4.4.1. Apple Fruit Transformation

For apple fruit transformation, Agrobacterium harboring vector pICH20155 (5′ module), pICH31070 (3′ module), and pICH14011 (integrase module) were separately inoculated. Overnight cultures of Agrobacterium cells were centrifuged, and then three modules were mixed in an infiltration buffer with an equal ratio. Apple fruits were transformed with a mixture of resuspended Agrobacterium cells using the vacuum-infiltration method [36]. The infiltrated fruits were divided into five groups: (1) MOCK; Agroinfiltrated with empty vector as control, (2) CHS∷31070, (3) ANS∷31070, (4) UFGT1∷31070, and (5) MIX: (CHS∷31070, ANS∷31070, and UFGT1∷31070). After the agroinfiltration, the fruit-outer surfaces were blotted to several layers of tissue paper to remove residual Agrobacterium and then kept overnight in darkness.

#### 4.4.2. Rice Callus Transformation

For the transformation of binary vector CHS∷1300 into embryogenic rice callus, the overnight cultured of transformed *A. tumefaciens* LBA4404 was suspended for rice callus transformation as previously studied [51]. In brief, the overnight culture of the transformed *A. tumefaciens* LBA4404 harboring CHS∷1300 vector was centrifuged at 4000× *g* for 15 min to collect the cells. The pellet of Agrobacterium cells was suspended in sterile water and centrifuged at 4000× *g* for 5 min. This step was repeated twice to remove any residue of the antibiotics (Kanamycin and Ri-famycin), which were added to the culture media during Agrobacterium cultures. The cell pellet was resuspended in an N6CI liquid medium supplemented with 200 µM of acetosyringone. The Agrobacterium was adjusted to a final OD600 of 0.5 and then was used for infection to rice embryogenic calli. The rice embryogenic callus was detached from the germinated seeds, which were obtained via in vitro culture (Section 4.1.2) and were used for plant transformation using the Agrobacterium-mediated transformation method [58]. The Agrobacterium-infected rice calli were then placed onto co-culture medium N6CO (N6CO = N6CI plus 1 g/L casamino acids, 10 g/L glucose, and 100 µM acetosyringone, pH 5.7) and cultured for a period of 3–5 days in darkness at 28 °C. After the co-culture period, the explants were repeatedly washed 3–4 times with sterile distilled to eliminate any excess Agrobacterium presence. The last rinse involved sterile distilled water mixed with 500 mg/L cefotaxime, and this was carried out for 10 min with gentle shaking. After washing, the explants were gently dried on sterile 3M filter paper before being transferred to the selection medium N6SE (N6SE = N6CI plus 250 mg/L cefotaxime, 50 mg/L hygromycin B, pH 5.7). Following this, transgenic rice calli were produced and selected using a medium containing hygromycin B. Regular subculturing and propagation of the transgenic rice callus were performed every 3–4 weeks. The entire in vitro cultivation process occurred within a controlled environment featuring a 16 h/8 h (light/dark) photoperiod, a light intensity of 100 μM.m^−2^.s^−1^, and a constant temperature of 25 °C.

### 4.5. Apple Fruit Sunlight Treatments

The infiltrated fruits of the five groups (120 fruits/group) were further divided into two subgroups (exposed and gagged) based on different light treatments, with 60 fruits per subgroup. Half of the fruits in each group were exposed to LED light with a 16 h/8 h (light/dark) photoperiod and light intensity 200 μM·m^−2^·s^−1^. The fruits were rotated every day to ensure both sides (upside and downside) of the fruit received optimal light. The other half of the fruits in each group were protected from light by bagging. The temperature was maintained constantly at 25 °C during treatments by keeping the fruits in a culture room. After two-day intervals, fruits in each subgroup were recorded for fruit coloration, and then 15 fruits were randomly collected and peeled for RNA extraction and pigment content analysis.

### 4.6. Measurements of Fruit Color and Fruit Quality

The fruit color and fruit quality were measured as previously reported [31]. For color properties, the peel of fruits was measured three times in the equatorial region of each fruit using a chromameter (CR-400, Konica Minolta, Japan) and expressed as a Hunter value. Fruit firmness was measured using a digital fruit firmness tester (TR Turoni, Forlì FC, Italy) at three random positions on the equatorial region of each fruit after peeling, expressed in newtons (N). For the measurement of soluble solids content (SSC), extracted juice was measured using a pocket refractometer (PAL-1, Atago, Tokyo, Japan) and expressed as °Brix. The total acidity of extracted juice was titrated using 0.1 N NaOH.

### 4.7. Genomic DNA Isolation and Polymerase Chain Reaction (PCR) Analysis

The putative transgenic rice callus growth on the selection media was sampled for the extraction of genomic DNA (gDNA) using a DNeasy^®^ Plant Mini Kit (Cat. #69204; Qiagen, Hilden, Germany). Transgenic rice callus lines were confirmed by PCR analysis for the detection of the transgene. PCR was carried out in 20 μL of reaction volume, including 1 uL of gDNA, using the Maxime™ PCR PreMix (i-StarTaq) (Cat. #25167; Intron, Seongnam, Republic of Korea). The gDNA PCR was run as follows: initial denaturation at 95 °C/5 min, followed by 30 repeated cycles of (95 °C/45 s–58 °C/45 s–72 °C/45 s) and a final extension at 72 °C for 2 min. Integration of the T-DNA region into the rice genome was investigated employing specific primer sets (Table S2). The PCR bands of the target gene MdCHS and the selection gene hptII were visualized under ultraviolet light and analyzed using the Image LabTM program (Bio-Rad Laboratories, Inc., Hercules, CA, USA).

### 4.8. RNA Isolation and Quantification of Gene Expression

Total RNA was isolated from the peel of ‘RubyS’ apple fruit using the CTAB method [59]. Rice RNA was isolated from the transgenic calli using an RNA Plant Mini Kit (Cat. #74904, Qiagen, Germany) according to the manufacturer’s instructions. Contamination of genomic DNA in the RNA samples was removed by DNase treatment (TURBO DNA-free™ Kit, Cat. #AM1907, Invitrogen, Carlsbad, CA, USA). RNA concentration was measured using a UV spectrophotometer (BioDrop µLite, Biochrom, Cambridge, UK). Reverse transcription was conducted using a PrimeScript™ 1st Strand cDNA Synthesis Kit with oligo (dT) primer (PrimeScript™ 1st strand cDNA Synthesis Kit, Takara, Kusatsu, Japan).

Transcript expression was quantified by quantitative reverse transcription PCR (qRT–PCR) and RT–PCR. qRT–PCR was conducted using LightCycler 480 SYBR Green I Master Mix (Roche) on a LightCycler 480 II Real-Time PCR System (Roche Diagnostics, Mannheim, Germany). RT–PCR was run in a total of 20 μL reaction volume with 1μL of cDNA as templates using a Maxime™ PCR PreMix. The primer sequences for the qRT-PCR and RT–PCR are listed in Table S2. Transcript expression levels in apple fruits were calculated by normalizing to the reference gene *MDP0000336547* [60]. The expression levels of *MdCHS* in transgenic rice calli were normalized to a rice reference gene *OsUbi1*.

### 4.9. Pigment Content Analysis

Samples collected from the apple peel and rice calli were ground to a fine powder in liquid nitrogen using a mortar and pestle. Anthocyanin content was calculated using the pH differential method [61]. Carotenoid content was determined using an HPLC system. Pigment content analysis was described as previously studied [36]. Accumulation of anthocyanin and carotenoid contents were determined as cyanidin-3-glucoside and β-carotene equivalents, respectively.

### 4.10. Data Analysis

The data were analyzed using Microsoft^®^ Excel^®^ for Microsoft 365 MSO (Version 2211 Build 16.0.15831.20098) (Microsoft Corp., Redmond, WA, USA) and IBM SPSS Statistics 23 software (IBM Corp., Armonk, NY, USA). Tukey’s test was used to analyze the difference between treatment groups. Pearson’s correlation coefficient test was used to evaluate the relationship between anthocyanin contents and gene expressions. The results were expressed as mean ± SD. For data on the expression levels of anthocyanin-related genes, error bars represent the SD of three biological replicates with seven technical replicates for each biological replicate. The values of *p* < 0.05 and <0.01 were considered statistically significant.

## 5. Conclusions

We investigated the roles of environmental (light) and transgenic factors (transient expression of three anthocyanin biosynthesis genes) in controlling the coloration and pigment content accumulation in apple fruit. Light, together with the transient expression of anthocyanin biosynthesis genes, showed synergistic effects on different coloration patterns, transcript expression levels, and pigment content accumulation in apple fruit. Infiltrated apples with transient expression of these genes using a TMV-based pro-vectors system and exposed to light showed a significantly higher expression of anthocyanin synthesis-related genes and a significantly greater accumulation of pigment content compared to the control. Furthermore, the important role of transgenic factors in the regulation of anthocyanin accumulation was also evaluated via stable expression of the anthocyanin biosynthesis gene in transgenic rice calli. Taken together, environmental and genetic factors have synergistic effects on the regulation of anthocyanin accumulation in plants. We can interfere with the anthocyanin synthesis pathway to induce anthocyanin accumulation in plant tissues via a non-transgenic (using environmental factors) or/and a transgenic (genetic manipulation of genes) approach. These findings contribute to our understanding of gene regulation and metabolic engineering in plants and could have implications for crop improvement and the production of specialized metabolites with potential health and commercial benefits.

## Data Availability

Data are contained within the article and Supplementary Materials.

## Supplementary Materials

The following supporting information can be downloaded at https://www.mdpi.com/article/10.3390/ijms241612946/s1.
