# Supplementary material for: The Synergistic Effects of Environmental and Genetic Factors on the Regulation of Anthocyanin Accumulation in Plant Tissues
Source: Int J Mol Sci. 2023 Aug 18;24(16):12946. doi: 10.3390/ijms241612946 (PMC10454628; doi:10.3390/ijms241612946)
Supplement: Supplementary file 1 [file ijms-24-12946-s001.zip › ijms-2555685-supplementary.pdf]

Article

Synergistic Effects of Environmental and Genetic Factors on Regulation of Anthocyanin Accumulation in Plant Tissues

Van Giap Do*, Youngsuk Lee, Jeong-Hee Kim, Young-Soon Kwon, Jong-Taek Park, Sangjin Yang, Juhyeon Park, Nay Myo Win, Seonae Kim*

Supplementary Table:

- Table S1. Fruit characteristic and colorimetric coordinates of 'RubyS' before treatment
- Table S2. Sequences of primers used for qRT-PCR and RT-PCR

Supplementary Figure:

- Figure S1. DNA sequence (CDS) of the three anthocyanin biosynthesis-related genes (*MdCHS*, *MdANS*, and *MdUFGT1*) cloned in this study
- Figure S2. A packing vector pMG-Kan for subcloning the synthesized CDS of three anthocyanin biosynthesis-related genes *(MdCHS, MdANS, and MdUFGT1)*.
- Figure S3. Vector construction of *MdCHS* gene in the packing vector pMG-Kan
- Figure S4. Vector construction of *MdANS* gene in the packing vector pMG-Kan
- Figure S5. Vector construction of *MdUFGT1* gene in the packing vector pMG-Kan
- Figure S6. Gene cloning and Agrobacterium transformation of *MdCHS* gene for transient overexpression
- Figure S7. Gene cloning and Agrobacterium transformation of *MdANS* gene for transient overexpression
- Figure S8. Gene cloning and Agrobacterium transformation of *MdUFGT1* gene for transient overexpression
- Figure S9. Gene cloning and Agrobacterium transformation of *MdCHS* gene for stable overexpression
- Figure S10. Expression profile of anthocyanin biosynthesis-related genes in the Agroinfiltrated leaves of *Nicotiana benthamiana*.

Table S1. Fruit characteristic and colorimetric of 'RubyS' before treatment

Group	Fruit characteristics							Colorimetric coordinates		
	Weight (g)	Vertical length (cm)	Horizontal length (cm)	Firmness (N)	SSC (°Brix)	Acidity (%)	Starch index	L	a	b
MOCK	74.0±7.6[a]	53.9±3.6[a]	57.1±3.8[a]	44.79±7.74[a]	12.3±0.40[a]	1.50±0.11[a]	1.53[a]	67.39±1.93[a]	-6.64±1.12[a]	28.63±0.51[a]
CHS::31070	75.0±7.11[a]	53.7±3.8[a]	56.6±2.5[a]	43.89±7.11[a]	12.4±0.47[a]	1.50±0.08[a]	1.53[a]	65.59±1.88[a]	-7.32±1.87[a]	27.55±0.92[a]
ANS::31070	75.6±5.5[a]	52.2±2.6[a]	55.9±3.4[a]	44.12±5.83[a]	12.1±0.35[a]	1.50±0.05[a]	1.46[a]	66.49±2.07[a]	-6.39±2.16[a]	27.66±0.90[a]
UFGT::31070	74.3±5.2[a]	51.8±3.0[a]	55.1±2.6[a]	42.52±4.88[a]	12.0±0.32[a]	1.50±0.02[a]	1.57[a]	66.39±2.88[a]	-6.99±2.78[a]	27.71±1.04[a]
MIX	75.7±6.1[a]	52.9±2.8[a]	57.1±3.3[a]	41.12±6.57[a]	12.2±0.45[a]	1.51±0.07[a]	1.60[a]	66.47±3.73[a]	-6.23±2.20[a]	27.32±1.02[a]

Data are means ± SD of three replications. Different letters above the bars indicate significant ($P < 0.05$) differences according to the t-test.

Table S2. Sequences of primers used in gDNA PCR and qRT-PCR

Gene	Encoded protein		Primer sequence (5′-3′)	Used for
MdCHS	Chalcone synthase	**F**	GCTCTAGAATGGTTACAGTCGAGGAAGTTCG	gDNA PCR (in rice callus)
		**R**	GGTCTCTCAGTGATGGTGATGGTGATGAGC	
HPTII	Hygromycin phosphotransferase	**F**	CTCGGAGGGCGAAGAATCTC	
		**R**	CAATGACCGCTGTTATGCGG	
MdCHS	Chalcone synthase	**F**	GGACTGGAACTCACTCTTC	qRT-PCR
		**R**	GCCGTAATCTGACAACAC	
MdANS	Anthocyanidin synthase	**F**	GCTGGAGAAAGAAGTTGG	
		**R**	GGAGGATGAAGGTGAGTG	
MdUFGT	UDP-glucose: flavonoid-3-O-glucosyl transferase	**F**	CAACATCCAAGGTCTCTC	
		**R**	GTCCCATCTGAAGTAGCA	
OsUbi1	Polyubiquitin1	**F**	CACGGTTCAACAACATCCAG	
		**R**	TGAAGACCCTGACTGGGAAG	
NbAct	Actin	**F**	CGAGCGGGAAATTGTTAGGG	
		**R**	CGTAGCTCTTCTCCACGGAT	
*Tnos**	Nopaline synthase terminator	**F**	CATGTAATGCATGACGTTATTTATG	
		**R**	TTGTTTTCTATCGCGTATTAAATGT	

*Tnos**: This primer was selected from a previous report by the EU, which was used as a reference method for the detection of *Nopaline synthase* terminator in GMO crops. See the detail at https://gmo-crl.jrc.ec.europa.eu/gmomethods/docs/QL-ELE-00-011.pdf

Supplementary Figure S1:

1/ *Malus x domestica* MdCHS mRNA for chalcone synthase, complete cds
GenBank: AB074485.1
Gene: 1..1176
CDS: 1..1176

[5′ BsaI-XbaI_MdCHS_6HisTag-Stop-BsaI-Acc65I/KpnI 3′]

GGTCTCTCTAGAATGGTTACAGTCGAGGAAGTTCGCAAGGCTCAACGGGCGGAGGGTCCAGCCACAGTCATGG
CCATCGGGACAGCAACTCCTTCCAACTGTGTGGATCAGGCTACCTACCCCGACTACTACTTTCGTATCACCAA
CAGCGAGCACAAGGTTGAGCTCAAAGAAAAATTCCAGCGCATGTGCGACAAATCTATGATCAAGAAACGTTAT
ATGTACTTGACTGAAGAAATTTTAAAAGAGAACCCAAGTGTGTGCGAGTACATGGCTCCTTCAATTGATGCAA
GGCAGGACATGGTGGTTGTGGAAGTCCCAAAACTTGGCAAAGAGGCTGCCATCAAAGCCATCAAGGAATGGGG
ACAGCCCAAGTCCAAAATCACCCACTTGGTCTTTTGCACCACCAGCGGTGTCGACATGCCTGGAGCCGACTAC
CAACTCACCAAGCTCTTGGGCCTCCGCCCCTCCGTCAAGCGCCTCATGATGTACCAACAAGGGTGCTTCGCCG
GTGGGACGGTCCTCCGTTTGGCCAAGGACTTGGCCGAAAACAACAAGGGTGCACGTGTTCTTGTTGTGTGCTC
TGAGATCACCGCGGTTACCTTCCGAGGGCCTAGTGACACCCACCTTGATAGTCTTGTGGGCCAAGCTTTGTTT
GGCGACGGTGCAGCGGCCGTCATCATTGGTGCGGATCCAGTGCCCGAAGTCGAGAAGCCCTTGTTTGAATTGG
TGTCGGCGGCACAAACCATTCTCCCCGACAGTGATGGGGCTATCGACGGACATCTCCGTGAAGTAGGGCTTAC
ATTTCACCTTCTCAAGGATGTTCCCGGACTTATTTCGAAGAACATCGAAAAGAGCCTTAACGAGGCCTTCAAG
CCTATTGGGATTTCGGACTGGAACTCGCTCTTCTGGATTGCACACCCAGGTGGCCCTGCTATTCTGGACCAAG
TAGAGGCCAAGTTGGCACTGAAGCCGGAGAAACTAGAAGCAACAAGGCAAGTGCTGTCGGATTACGGTAACAT
GTCGAGTGCGTGTGTGCTTTTTATTTTGGACGAGGTCAGGAGGAAGTCCGCCGAGAAAGGACTCAAAACGACC
GGGGAGGGACTGGAGTGGGGTGTGCTTTTCGGATTTGGGCCCGGCCTCACGGTGGAAACCGTCGTGCTTCACA
GCGTGGGTTTAACGGCTCATCACCATCACCATCACTGAGGTCTCGGTACCGG

2/ *Malus domestica* anthocyanidin synthase (ANS)mRNA, complete cds
GenBank: AF117269.1
Gene: 1..1413
CDS: 90..1163

[5′ BsaI-XbaI-BamHI_MdANS_6HisTag-Stop-BsaI-Acc65I/KpnI 3′]

GGTCTCTCTAGAGGATCCATGGTGAGCTCTGATTCAGTGAATTCAAGGGTTGAAACCTTGGCCGGCAGTGGAA
TCTCAACCATCCCAAAAGAGTACATCAGACCTAAAGATGAGCTCGTAAACATTGGTGACATCTTCGAACAAGA
GAAGAACAACGAAGGGCCTCAAGTTCCCACCATCGATTTGAAGGAGATAGAGTCTGATAACGAAAAAGTGAGA
GCAAAATGCAGGGAGAAGTTGAAGAAGGCAGCTGTGGACTGGGGTGTCATGCACCTTGTGAACCATGGCATCT
CCGACGAGCTCATGGACAAGGTCAGGAAGGCCGGTAAGGCCTTCTTTGACCTTCCCATTGAGCAGAAGGAGAA
GTATGCCAATGACCAGGCCTCTGGTAAGATTCAAGGCTATGGAAGCAAGCTTGCAAACAATGCATCTGGGCAG
CTTGAGTGGGAGGACTACTTCTTCCACTGTGTATACCCAGAGGACAAGCGTGACTTGTCAATTTGGCCTCAAA
CACCTGCTGATTACATTGAGGCAACCGCCGAGTATGCTAAGCAATTGAGGGAGCTAGCAACCAAGGTACTGAA
AGTTCTGTCACTTGGCTTGGGATTGGATGAAGGGAGGCTGGAGAAAGAAGTTGGTGGACTTGAAGAGCTCCTC
TTGCAAATGAAAATCAACTACTACCCAAAATGCCCTCAGCCGGAGCTTGCACTTGGTGTTGAAGCTCACACTG
ACGTGAGTGCACTCACCTTCATCCTCCACAACATGGTTCCTGGCCTGCAGCTTTTCTATGAAGGAAAGTGGGT
CACTGCCAAGTGCGTTCCAAATTCCATCGTCATGCACATTGGGGACACACTTGAGATTTTGAGCAATGGGAAG
TACAAAAGTATACTCCACAGGGGCATGGTGAACAAGGAAAAGGTGAGGATTTCATGGGCTGTTTTCTGTGAGC
CACCAAAGGAGAAGATCATCCTTAAGCCACTGCCGGAAACCGTGTCTGAGGACGAGCCGGCAATGTTCCCACC
ACGAACTTTTGCTGAGCACATTCAGCACAAGTTGTTCAGGAAGAGCCAAGAGGCTTTGCTCCCCAAGCATCAC
CATCACCATCACTGAGGTCTCGGTACCGG

3/ *Malus domestica* UDP glucose:flavonoid 3-O-glucosyl transferase (UFGT1)mRNA, complete cds

GenBank: AF117267.1
Gene: 1..1819
CDS: 72..1523

[5′ BsaI-XbaI-BamHI_MdUFGT1_6HisTag-Stop-BsaI-Acc65I/KpnI 3′]

GGTCTCTCTAGAGGATCCATGGCAGCGCCGCTGCCCATCGAAATCGAACCATCATCAACTAATGGTCAACCCC
ATCTCGCCGACGCCTACAACCGTCACGTGGCTGTCGTAGCCTTCCCTTTCACTAGCCATGCAAGCGCCTTGCT
TGAAACCGTGCGCCGCCTAGCCACCGCCCTTCCAAACACTCTCTTCTCGTTCTTCAGCACTTCAAAATCCAAC
AGCTCTCTCTTTTCCAACAACAGCATTGATAACATGCCGCGTAACATAAGGGTGTACGATGTGGCTGACGGGG
TGCCGGAGGGGTACGTTTTCGTGGGCAAGCCGCAGGAGGACATAGAGCTCTTCATGAATGCCGCACCGGAAAA
CATCCGGAGGAGCTTAGACGCTTCCGTGGCGGACATCGGGAAGCAGATCAGCTGCTTGATCACCGACGCCTTC
CTTTGGTTTGGAGTCCACTTGGCTGACGAGTTGGGAGTGCCTTGGGTCACTTTCTGGATCTCCGGACTCAAAT
CCCTCTCCGTTCATGTGCATACTGATCTCATCCGCGACACTATTGGAACTCAAGGCATTACAGGTCGTGAAAA
CGACCTCATCGTCGACAAAAATGTTAACATCCAAGGACTCTCCAATGTACGAATCAAAGACTTAGCGGAAGGA
GTCATTTTCGGAAACTTGGACTCGGTAATTTCCGGCATGCTACTTCAGATGGGACGGCTCCTCCCCCGTGCCA
CCGCAGTTTTCATGAACGGCTTCGAAGAATTGGAACTCCCCATACCAAACGACCTAAAGTCCAAAGTCAACAA
ACTCCTCAACGTAGGACCTTCCAACGTAGCATCCCCGCTGCCACCGCTGCCGCCATCAGATGCTTGCTTGTCA
TGGCTAGACAAGCAACAGGCTCCATCCTCCGTCGTGTACATAAGCTTCGGGACAGTGGCGAGCCCAGCGGAGA
AGGAGCAGATGGCAATAGCGGAGGCCCTGGAAGCCACCGGAGCACCCTTCTTGTGGTCTATCAAGGACAGCTG
CAAGACACCGTTGCTGAACGAGTTCTTGACAAAAACATTGTCAAAGCTGAACGGGATGGTGGTGCCGTGGGCT
CCACAGCCGCATGTACTGGCCCACGATTCGGTCGGAGCCTTCGTGTCGCATTGCGGCTGGAACTCGATAATGG
AGACTATAGCAGGACGGGTGCCCATGATTTGTAGGCCATATTTTGCAGACCAGAGGCTTAATGCAAGGATGGT
GGAGGAGGTGTTTGAGATCGGGGTAACCGTGGAGGATGGAGTTTTTACCAGGGAGGGGCTGGTAAAAAGCTTG
GAAGTGGTTTTGTCGCCTGAAAGTGGGAGGAAATTCAGAGACAATATAAAGAGGGTCAAACAACTGGCAGTAG
AGGCGGTTGGACCACAAGGGAGCTCCACTCGGAACTTCAAATCGCTGTTGGACATCGTATCAGGTTCCAATTA
TCAAGTACATCACCATCACCATCACTAGGGTCTCGGTACCGG

Figure S1. DNA sequence (CDS) of three anthocyanin biosynthesis-related genes (*MdCHS*, *MdANS*, and *MdUFGT1*) cloned in this study. They were synthesized containing restriction enzyme sites in colored for cloning purposes.

pMG-Kan Map

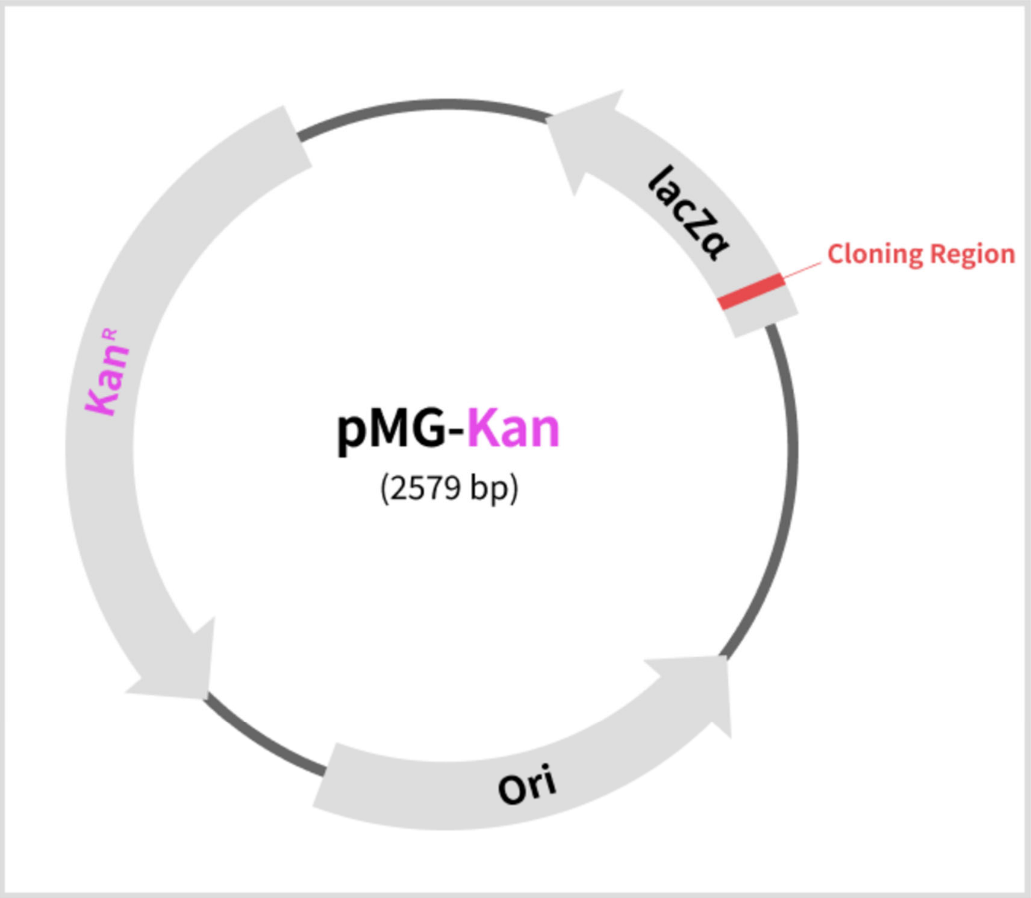

Cloning Region

GTTTTCCCAGTCACGACGCTGTAAAACGAC
GGCCAGTGAATTACTTGAAGGTACTTCTCT
AATACATCTTGAAATCGGGTCCC/GGGCTC
TACGAGAGCACAGTCCGGCGTGCAGAGAT
GGCGTAATCATGGTCATAGCTGTTTCCTGT
GTGAAATTGTTATCCGC

Sequencing Primer

M13F-pUC	GTTTTCCCAGTCACGAC
M13R	GCGGATAACAATTTCACACAGG

Figure S2. A packing vector pMG-Kan for subcloning the synthesized CDS of three anthocyanin biosynthesis-related genes(*MdCHS*, *MdANS*, and *MdUFGT1*).

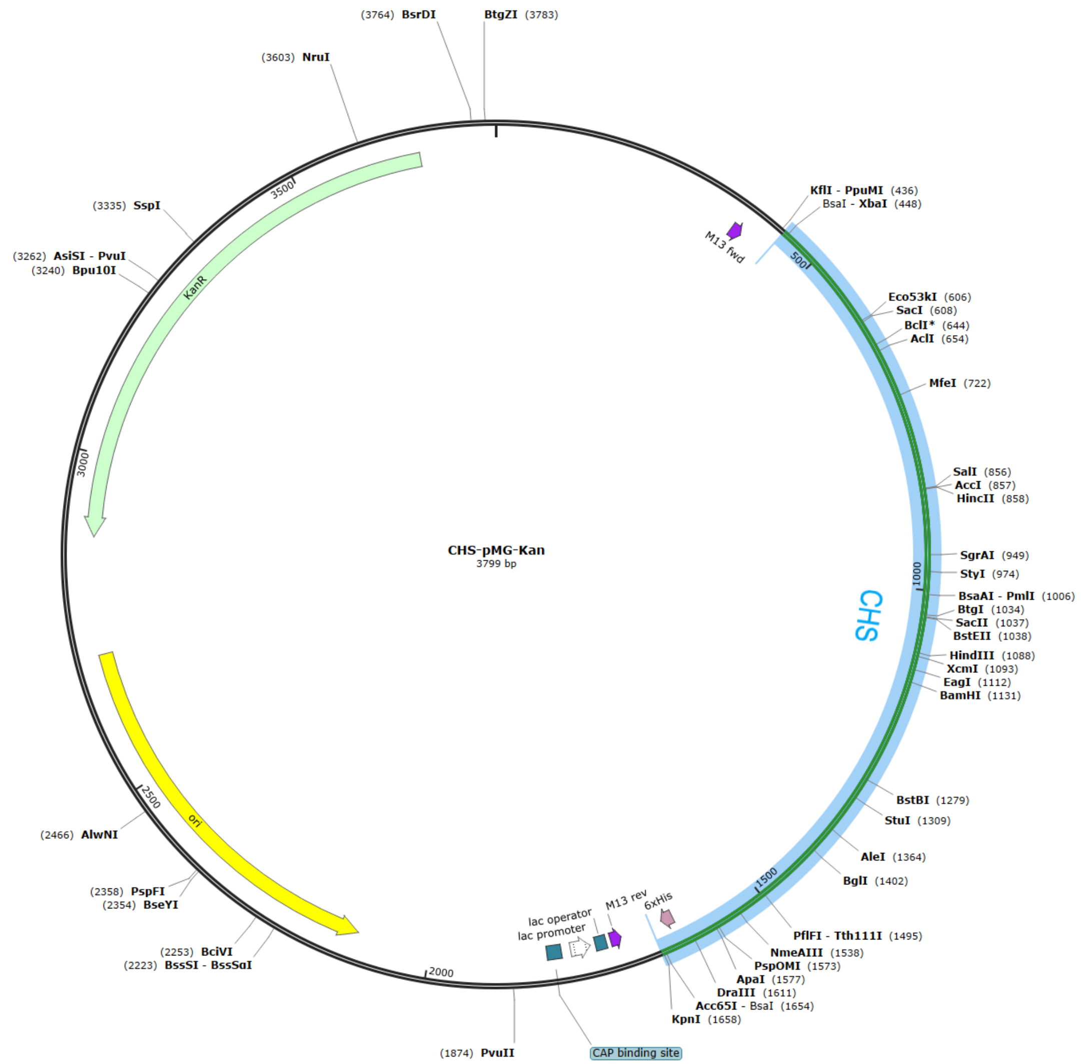

Figure S3. Vector construction of *MdCHS* gene in the packing vector pMG-Kan.

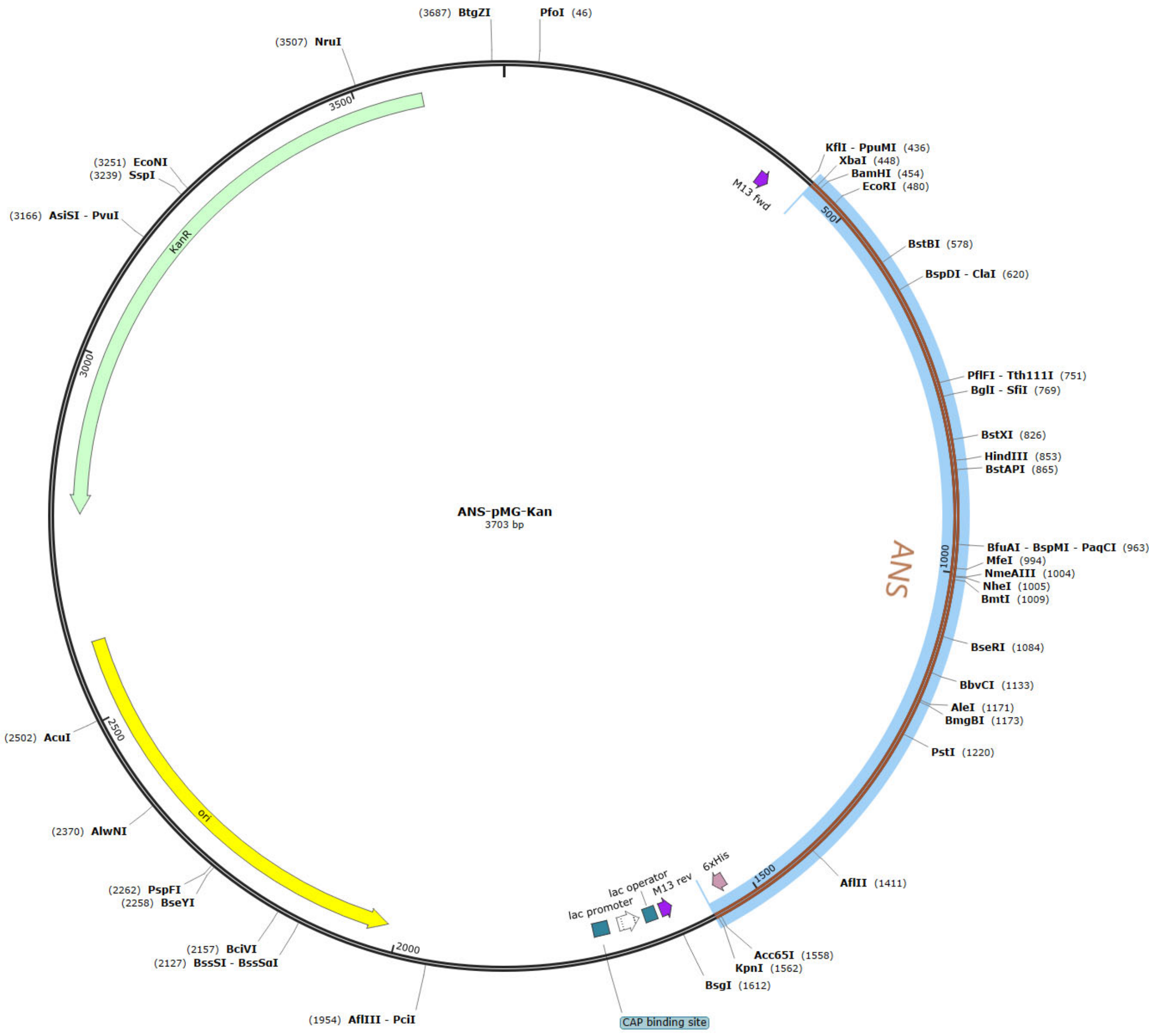

Figure S4. Vector construction of *MdANS* gene in the packing vector pMG-Kan.

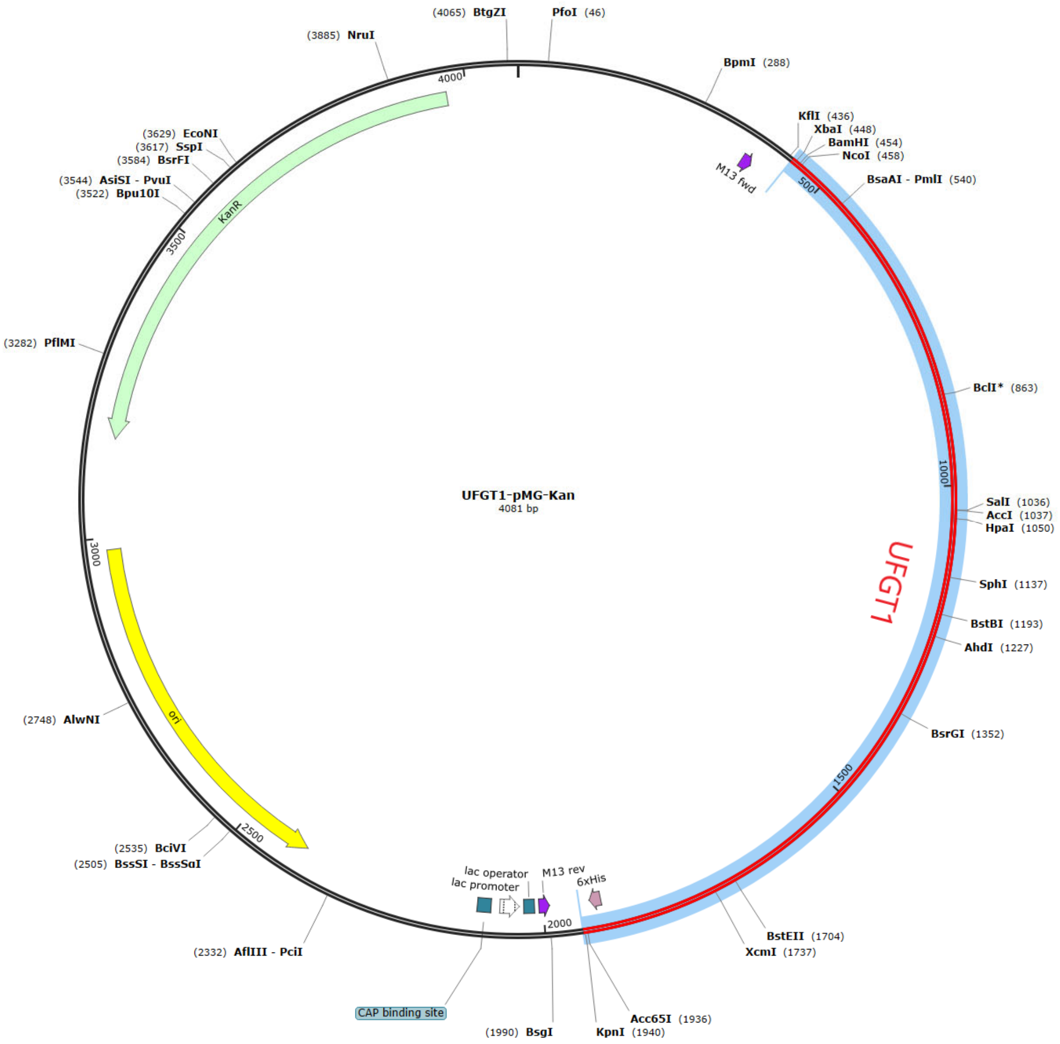

Figure S5. Vector construction of *MdUFGT1* in the packing vector pMG-Kan.

Chalcone synthase (CHS)

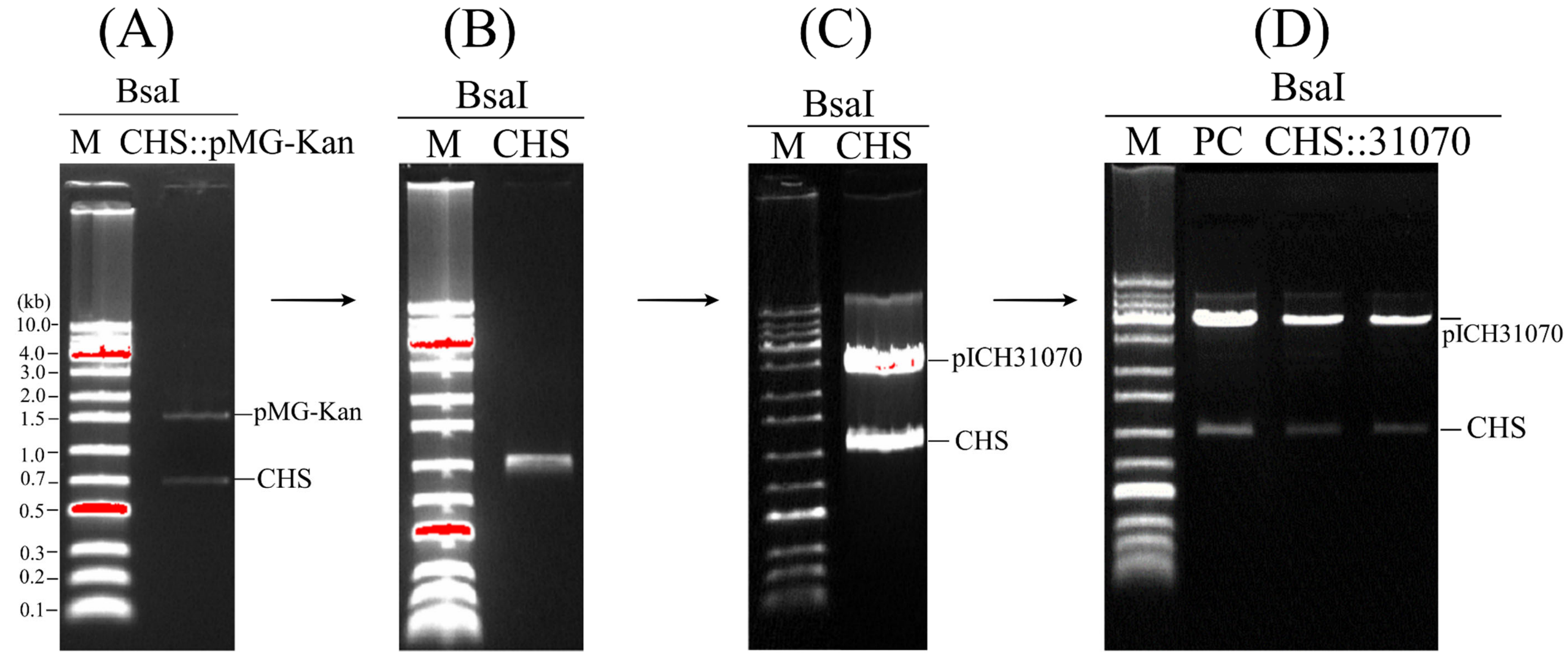

Figure S6. The synthesized CDS of *MdCHS* containing a BsaI site at the 5'end and the 3'end were subcloned on packing vector pMG-Kan was digested with BsaI (A) and then the digested fragments were eluted and cleaned up from agarose gel using the fragment DNA purification kit (iNtRON Biotechnology) before cloning into the plant expression vector pICH31070, resulted CHS::pICH30170. The constructed expression vector CHS::pICH30170 was transformed into *E.coli* DH5α then was confirmed by BsaI enzyme digestion (C). The expression vector was transformed into *A. tumefaciens* EHA105 and then its plasmid DNA was isolated and confirmed by BsaI enzyme digestion (D). M is 1kb plus DNA ladder (Cat.#A738, Dyne Bio Inc. Seongnam-si, Korea).

Anthocyanidin Synthase (ANS)

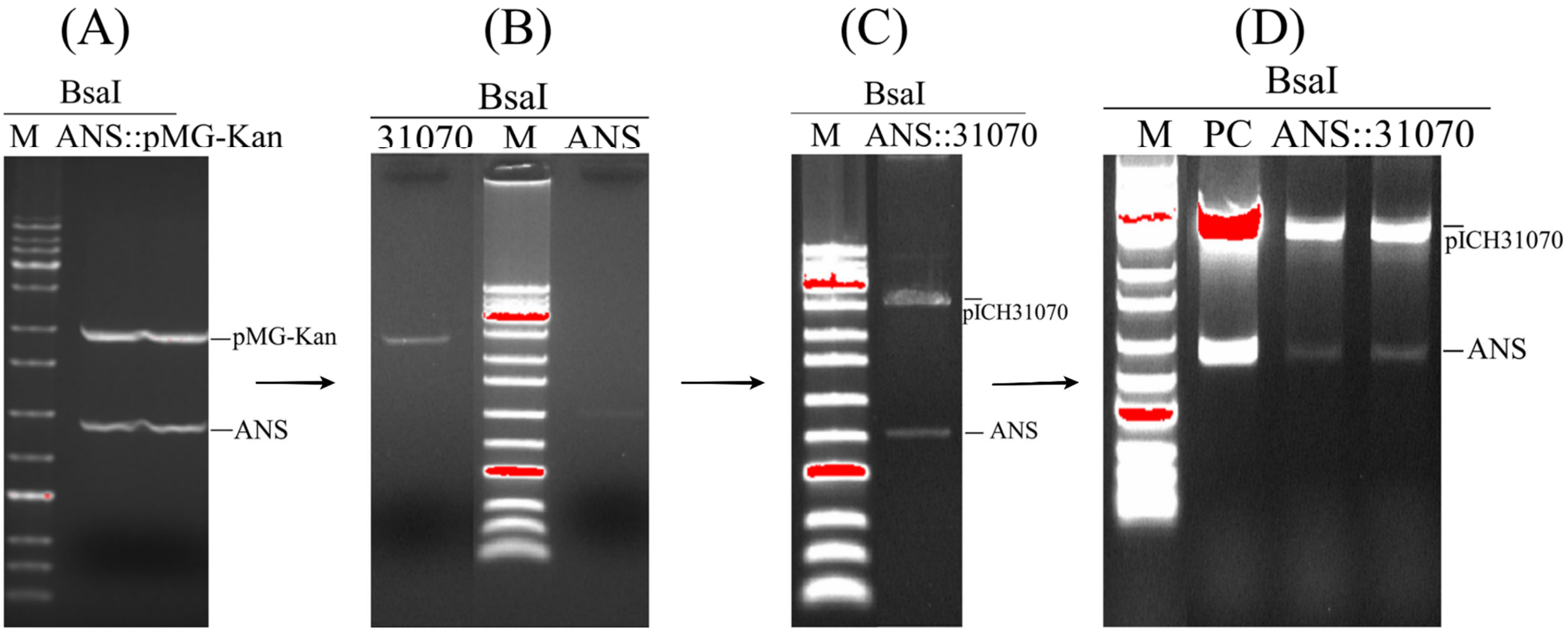

Figure S7. The synthesized CDS of *MdANS* containing a BsaI site at the 5′end and the 3′end were subcloned on packing vector pMG-Kan was digested with BsaI (A) and then the digested fragments were eluted and cleaned up from agarose gel using the fragment DNA purification kit (iNtRON Biotechnology) before cloning into the plant expression vector pICH31070, resulted ANS::pICH30170. The constructed expression vector ANS::pICH30170 was transformed into *E.coli* DH5*α* then was confirmed by BsaI enzyme digestion (C). The expression vector was transformed into *A. tumefaciens* EHA105 and then its plasmid DNA was isolated and confirmed by BsaI enzyme digestion (D).

UDP-Glucose: Flavonoid 3-O-Glucosyl Transferase (UFGT)

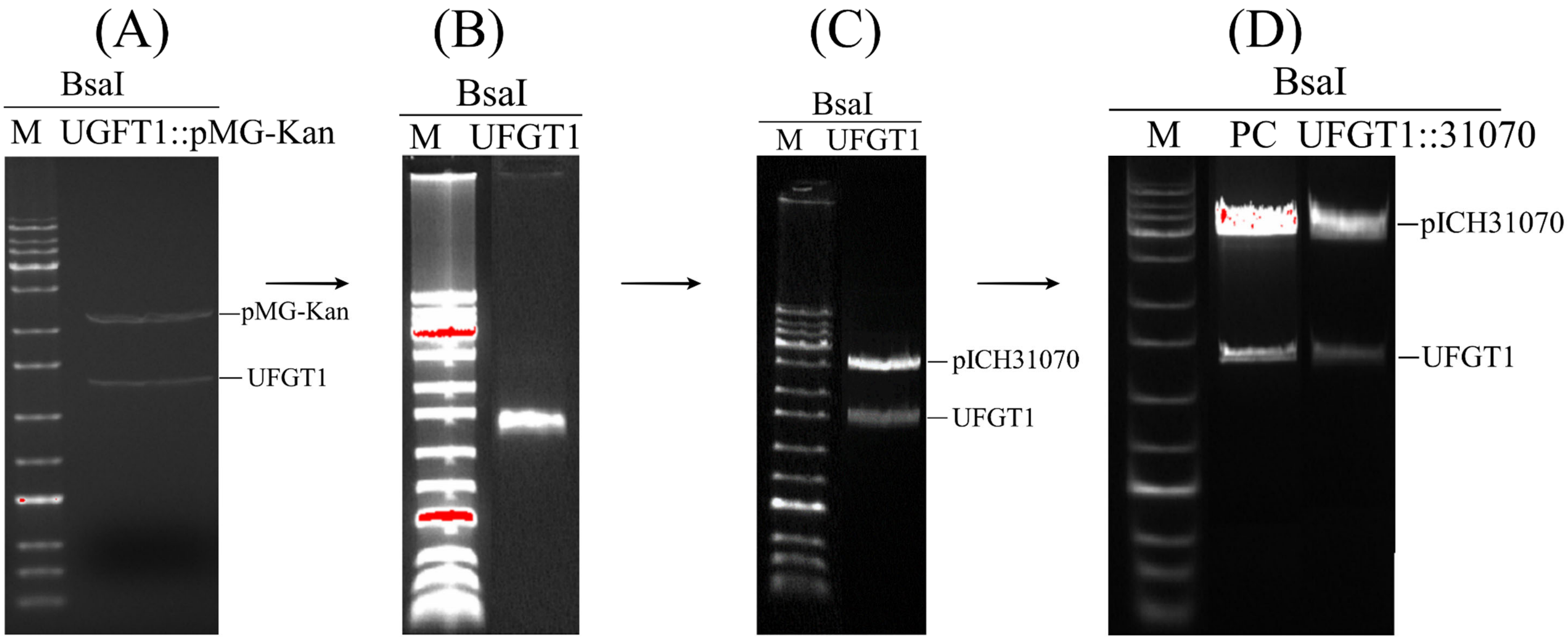

Figure S8. The synthesized CDS of *MdUFGT1* containing a BsaI site at the 5′end and the 3′end were subcloned on packing vector pMG-Kan was digested with BsaI (A) and then the digested fragments were eluted and cleaned up from agarose gel using the fragment DNA purification kit (iNtRON Biotechnology) before cloning into the plant expression vector pICH31070, resulted UFGT1::pICH30170. The constructed expression vector UFGT1::pICH30170 was transformed into *E.coli* DH5*α* then was confirmed by BsaI enzyme digestion (C). The expression vector was transformed into *A. tumefaciens* EHA105 and then its plasmid DNA was isolated and confirmed by BsaI enzyme digestion (D).

Chalcone synthase (CHS) for stable overexpression

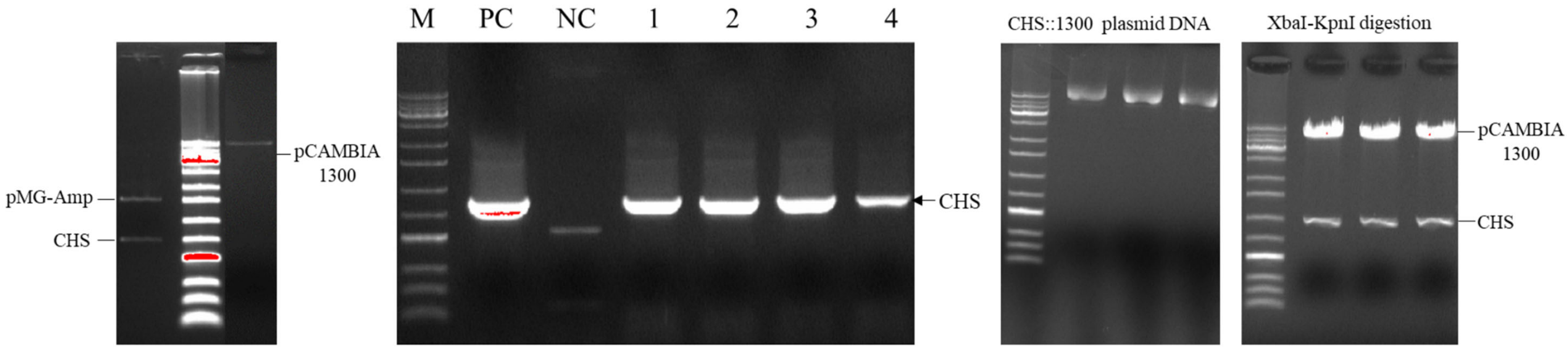

(A) (B) (C)

Figure S9. The synthesized CDS of *MdCHS* containing a XbaI site at the 5′-end and a KpnI site at the 3′-end were subcloned on packing vector pMG-Amp was digested with XbaI and KpnI (A) and then the digested fragments were eluted and cleaned up from agarose gel using the fragment DNA purification kit (iNtRON Biotechnology) before cloning into the plant expression vector pMYD320. The constructed expression vector was transformed into *E.coli* DH5α and then was confirmed by colony PCR (B). The plasmid DNA of transformed *A. tumefaciens* LBA4404 and XbaI/KpnI double enzyme digestion of its (C).

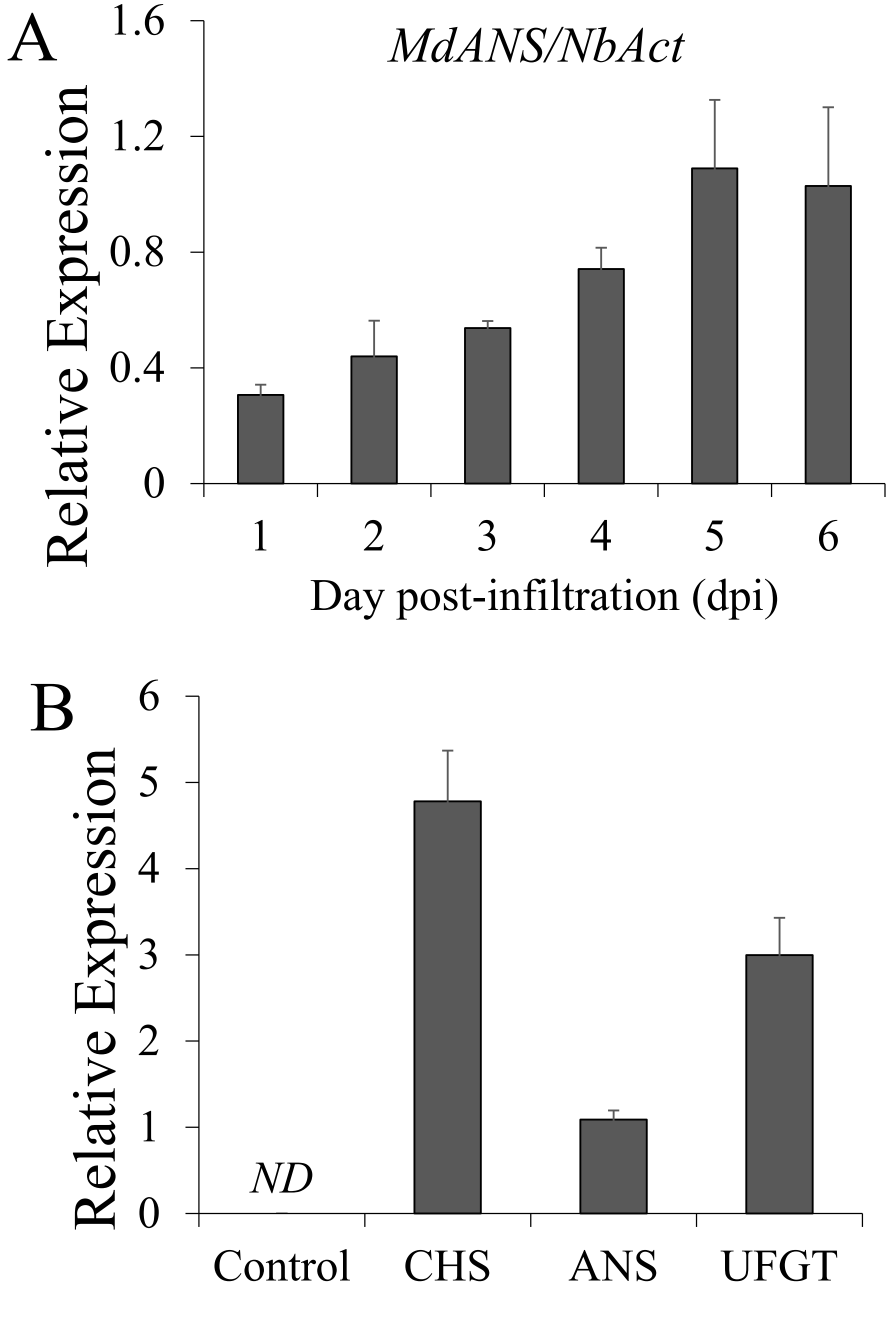

Figure S10. Expression profile of anthocyanin biosynthesis-related genes in the Agroinfiltrated leaves of *Nicotiana benthamiana*. (A) The transcript expression of *MdANS* was analyzed at different time course (1-6 dpi). (B) The expression profile of three anthocyanin biosynthesis-related genes (*MdCHS*, *MdANS*, and *MdUFGT*) at 5 dpi. Tobacco levaes were infiltrated with Agrobacterium OD600 = 0.6 and the transcript expression was calculated via normalization to the reference gene *NtAct*. Data are presented as mean ± SD (n = 3) of three biological replicates. *ND* refers to non-detectable.
